# Redistribution of the chromatin remodeler Brg1 directs smooth muscle–derived adventitial progenitor–to–myofibroblast differentiation and vascular fibrosis

**DOI:** 10.1172/jci.insight.164862

**Published:** 2023-05-08

**Authors:** Austin J. Jolly, Sizhao Lu, Allison M. Dubner, Keith A. Strand, Marie F. Mutryn, Aaron Pilotti-Riley, Etienne P. Danis, Raphael A. Nemenoff, Karen S. Moulton, Mark W. Majesky, Mary C.M. Weiser-Evans

**Affiliations:** 1 Department of Medicine, Division of Renal Diseases and Hypertension,; 2 Medical Scientist Training Program,; 3 School of Medicine, Consortium for Fibrosis Research and Translation,; 4 Department of Pharmacology,; 5 Cardiovascular Pulmonary Research Program, and; 6 Department of Medicine, Division of Cardiology, University of Colorado Anschutz Medical Campus, Aurora, Colorado, USA.; 7 Center for Developmental Biology & Regenerative Medicine, Seattle Children’s Research Institute, Seattle, Washington, USA.; 8 Departments of Pediatrics and Pathology, University of Washington, Seattle, Washington, USA.

**Keywords:** Vascular Biology, Adult stem cells, Epigenetics, Fibrosis

## Abstract

Vascular smooth muscle–derived Sca1^+^ adventitial progenitor (AdvSca1-SM) cells are tissue-resident, multipotent stem cells that contribute to progression of vascular remodeling and fibrosis. Upon acute vascular injury, AdvSca1-SM cells differentiate into myofibroblasts and are embedded in perivascular collagen and the extracellular matrix. While the phenotypic properties of AdvSca1-SM–derived myofibroblasts have been defined, the underlying epigenetic regulators driving the AdvSca1-SM–to–myofibroblast transition are unclear. We show that the chromatin remodeler *Smarca4*/Brg1 facilitates AdvSca1-SM myofibroblast differentiation. Brg1 mRNA and protein were upregulated in AdvSca1-SM cells after acute vascular injury, and pharmacological inhibition of Brg1 by the small molecule PFI-3 attenuated perivascular fibrosis and adventitial expansion. TGF-β_1_ stimulation of AdvSca1-SM cells in vitro reduced expression of stemness genes while inducing expression of myofibroblast genes that was associated with enhanced contractility; PFI blocked TGF-β_1_–induced phenotypic transition. Similarly, genetic knockdown of Brg1 in vivo reduced adventitial remodeling and fibrosis and reversed AdvSca1-SM–to–myofibroblast transition in vitro. Mechanistically, TGF-β_1_ promoted redistribution of Brg1 from distal intergenic sites of stemness genes and recruitment to promoter regions of myofibroblast-related genes, which was blocked by PFI-3. These data provide insight into epigenetic regulation of resident vascular progenitor cell differentiation and support that manipulating the AdvSca1-SM phenotype will provide antifibrotic clinical benefits.

## Graphical abstract

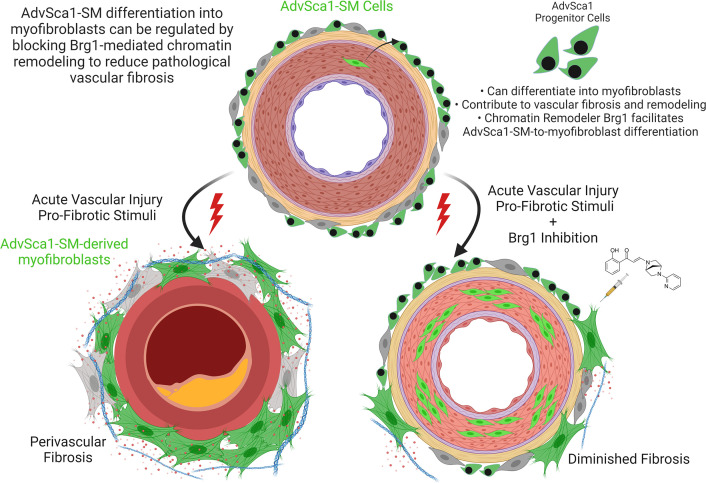

## Introduction

The vascular wall is a complex, multilayered tissue containing numerous cell populations that coordinate to maintain vessel homeostasis and regulate vascular remodeling in disease states. The outermost layer of major arteries, the tunica adventitia, is composed of pericytes, fibroblasts, adipocytes, WBCs, and resident progenitor/stem cells all supported by extracellular matrix, perivascular fat, and vasa vasorum (1–5). Adventitial remodeling occurs during chronic vascular disease or following acute vascular injury, as adventitial cells proliferate, secrete proinflammatory cytokines that recruit circulating leukocytes, and increase extracellular matrix deposition, leading to chronic vascular inflammation and stiffening (6, 7). Of the cell populations found within the adventitia, Stem cell antigen-1^+^ progenitor cells (AdvSca1 cells) have become a population of increased interest because these multipotent cells demonstrate remarkable heterogeneity with specific differentiation capabilities and are, therefore, likely important for pathological vascular remodeling and vascular repair (3). Using smooth muscle cell–specific lineage tracing and RNA-Seq, our group characterized a subpopulation of AdvSca1 cells that originate from mature smooth muscle cells (SMC) through an in situ reprogramming process (termed AdvSca1-SM cells) (8). Compared with other adventitial populations and mature SMCs, AdvSca1-SM cells exhibit a transcriptomic profile enriched for sonic hedgehog/Wnt/β-catenin signaling and extracellular matrix remodeling (8, 9). We generated and validated a tamoxifen-inducible AdvSca1-SM cell lineage mapping system to study the behavior of AdvSca1-SM cells in response to acute vascular injury. Our published data show that AdvSca1-SM cells differentiate into myofibroblasts, secrete numerous proremodeling factors including extracellular matrix proteins and proinflammatory cytokines, and contribute to perivascular fibrosis progression (9, 10). We and others also showed that AdvSca1-SM cells migrate to the media after vascular injury, where they can differentiate into mature SMCs and repair the vascular wall (9, 11). While the phenotypic changes of AdvSca1-SM cells in response to vascular insult have been described, the epigenetic regulation of the underlying gene expression influencing their differentiation is poorly understood.

Brahma-related gene 1 (*Smarca4*/Brg1) is a highly conserved protein catalyst for the switching/sucrose nonfermenting (SWI/SNF) complex, a chromatin remodeling machine originally discovered in yeast that displaces histones from tightly wound DNA, making the underlying DNA more accessible for transcription factors and other key proteins required for messenger RNA transcription (12, 13). The ATPase domain of the Brg1 protein is required for histone displacement, while the C-terminal bromodomain, a reader subunit that recognizes acetylated histone tails, facilitates recruitment of Brg1 to target sites throughout the genome. The bromodomain is also important for binding with other protein partners, including histone modifying enzymes (e.g., histone deacetylases, methyltransferases), nuclear receptors (e.g. glucocorticoid receptor), and transcription factors (e.g., Mef2D, β-catenin) (12, 14–16).

Brg1 activity is implicated in tissue fibrosis across many organs as well as in vascular development and remodeling (17). Li et al. showed that Brg1 expression is directly correlated with liver fibrosis in patients with cirrhosis and hepatocellular carcinoma and in mouse models of carbon tetrachloride–induced (CCl_4_-induced) liver fibrosis. Brg1-KO mice displayed decreased CCl_4_-mediated liver fibrosis as compared with WT animals (18). In a model of angiotensin II–mediated cardiac fibrosis, genetic ablation of Brg1 or pharmacologic inhibition of the bromodomain impaired vascular endothelial cell ability to undergo endothelial-to-mesenchymal transition. Brg1 depletion led to decreased cardiac interstitial fibrosis and preserved ejection fraction (19). Reexpression of Brg1 in Brg1-null pancreatic ductal carcinoma cells induced epithelial-to-mesenchymal transition characterized by expression of αSMA, vimentin, loss of the epithelial marker E-Cadherin, and a change in morphology to a more elongated, spindle-shaped form (20). Together, these studies illustrate profibrogenic functions for Brg1 across many organ systems in disease states.

The functions of Brg1 across the vascular wall are not completely known; however, a paper by Zhou et al. elucidated that mature aortic SMCs are dependent on Brg1 to maintain their contractile phenotype (21). They showed that Brg1 and its homolog Brm are required for mature aortic SMCs to sustain expression of genes that encode for contractile proteins, including smooth-muscle myosin heavy chain (SMMHC) and calponin. The authors also showed that the introduction of an ATPase-deficient Brg1 mutant (K798R missense mutation) to SMCs prevents activation of SMC gene expression by the myocardin-SRF complex. Cotransfection of myocardin and WT Brg1 into Brg1-deficient SW13 cells induced their responsiveness to myocardin (21). While these findings contribute to our understanding of Brg1 in mature SMCs, Brg1 function in AdvSca1-SM cells is unknown. AdvSca1-SM cells are major regulators of vascular remodeling and fibrosis, and investigating epigenetic mechanisms that underlie their differentiation yields the potential to manipulate their phenotype for clinical benefit. Here, we demonstrate that Brg1 facilitates AdvSca1-SM differentiation to myofibroblasts and that inhibiting Brg1 decreases injury-induced pathological vascular remodeling.

## Results

### Brg1 is upregulated in AdvSca1-SM cells after carotid ligation injury and in human hypertrophic left ventricular tissue.

**Figure 1:**
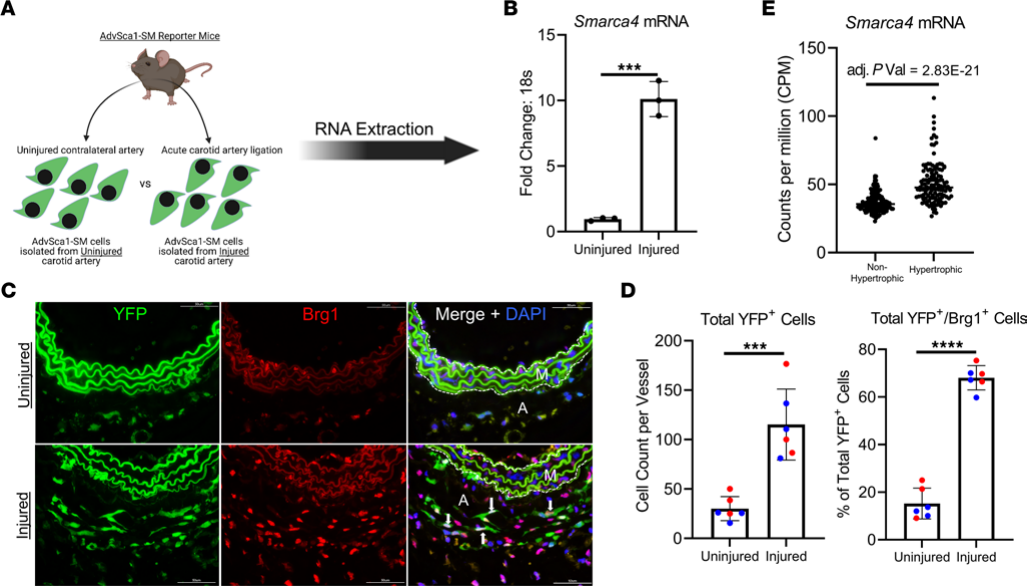
Carotid artery ligation increases Brg1 expression in AdvSca1-SM cells. (**A**) AdvSca1-SM cells were isolated from injured and uninjured carotid artery tissues from SM22α-Cre-YFP mice by FACS 3 days after ligation, and RNA extraction was performed. (**B**) *Smarca4* (Brg1) mRNA expression was assessed by qPCR. Data represent 3 independent biological replicates. A 2-tailed unpaired *t* test was performed to compare the means of the 2 experimental groups; ***P < 0.001. (**C**) Uninjured and injured carotid arteries were harvested from tamoxifen-treated *Gli1*-Cre^ERT^-YFP AdvSca1-SM reporter mice 3 days after ligation, embedded in OCT, and immunofluorescently stained for YFP (green) to identify AdvSca1-SM cells and for Brg1 (red). A, Adventitia; M, Media. Elastin autofluorescence is observed on the green and red channel. Scale bar: 50 μm. (**D**) Total YFP^+^ and YFP^+^/Brg1^+^ cells were counted from stained slides from each group. Data are shown as total number per high-magnification field. *n* = 6 (uninjured), *n* = 6 (injured). Blue data points represent male samples, and red data points represent female samples. A 2-tailed unpaired *t* test was performed to compare the means of the 2 experimental groups; ***P < 0.001, ****P < 0.0001. (**E**) *Smarca4* (Brg1) mRNA expression from human left ventricular tissue was obtained from the publicly available Genome Tissue Expression Database. The data were stratified by *NPPB* expression, nonhypertrophic tissue was defined as the lowest 30% of *NPPB* expression, and hypertrophic tissue was defined as the highest 30% of *NPPB* expression.

Carotid arterial ligation is a well-characterized model of acute vascular injury that results in neointima formation, adventitial expansion and fibrosis, and vascular inflammation (3, 9, 22–25). We previously showed that AdvSca1-SM cells contribute to adventitial remodeling by expanding and differentiating into myofibroblasts in response to carotid ligation (9). In our previous study, we performed unbiased RNA-Seq of AdvSca1-SM cells isolated from injured carotid arteries and compared them with RNA samples from AdvSca1-SM cells isolated from uninjured carotid arteries. In response to injury, AdvSca1-SM cells downregulated stemness-related genes, including *Ly6a/*Sca1, *Cd34*, *Gli1*, and *Klf4*, and upregulated genes related to a myofibroblast phenotype (e.g., *Acta2*, *Postn*, *Col1a1*, *Fn1*) and an inflammatory phenotype (e.g., *Ccl2*, *Tnfα*, *IL-1β*). Upon further analysis of the differentially expressed genes, we observed that *Smarca4*, the gene that encodes for the chromatin remodeling protein Brg1, was significantly upregulated by AdvSca1-SM cells in response to acute vascular injury (9). To validate this observation, we isolated AdvSca1-SM cells from ligated carotid arteries and from contralateral uninjured carotid arteries from SM22α-Cre ROSA-YFP mice 3 days after ligation surgery; RNA was extracted from these cell samples and subjected to quantitative PCR (qPCR) analysis (Figure 1A). SM22α-Cre mice were used to isolate AdvSca1-SM cells for all qPCR and other in vitro studies, since the yield of AdvSca1-SM sorted cells is higher from this model as compared with the AdvSca1-SM-specific reporter mice; in our previous study, we showed an identical gene signature of AdvSca1-SM cells from both systems (9). Compared with uninjured samples, *Smarca4* mRNA levels were elevated in AdvSca1-SM cells isolated from injured carotid arteries, consistent with the previous RNA-Seq data set (Figure 1B). To evaluate whether Brg1 protein expression was elevated after carotid ligation, histologic specimens were prepared from injured and uninjured carotid arteries from Gli1-Cre^ERT2^ ROSA-YFP AdvSca1-SM reporter mice 3 days after carotid ligation and examined by immunofluorescence microscopy (Figure 1C). Gli1-Cre^ERT2^ ROSA-YFP mice were used for all immunofluorescence lineage tracing studies because this tamoxifen-inducible model allows for controlled labeling specifically of the AdvSca1-SM population. Uninjured carotid arteries exhibited low Brg1 signal in the adventitia and media with less than 20% of YFP^+^ AdvSca1-SM cells expressing Brg1. Injured arteries exhibited increased overall numbers of YFP^+^ AdvSca1-SM–derived cells, and 70% of AdvSca1-SM cells coexpressed nuclear Brg1 (Figure 1D). Next, to add translational significance, we mined the publicly available human Genotype Expression Database (GTEx, https://gtexportal.org/home/) to examine *Smarca4* mRNA expression in human left ventricular tissue. We stratified the data set by expression of Natriuretic peptide B (*NPPB*), a clinical marker of heart failure and perivascular fibrosis, and defined hypertrophic hearts as the top 30% of *NPPB* expression and healthy hearts as the bottom 30% of *NPPB* expression. Consistent with *Smarca4* induction in response to vascular injury in the mouse, *Smarca4* expression was significantly increased in human hypertrophic left ventricular tissues as compared with nonhypertrophic left ventricular tissue (Figure 1E). Taken together, these results indicate that acute vascular injury induces Brg1 expression in AdvSca1-SM cells of the mouse carotid artery and that Brg1 is elevated in left ventricular tissue in patients with heart failure.

### Pharmacologic inhibition of the Brg1 bromodomain attenuates injury-induced pathological vascular remodeling at early and late stages.

**Figure 2:**
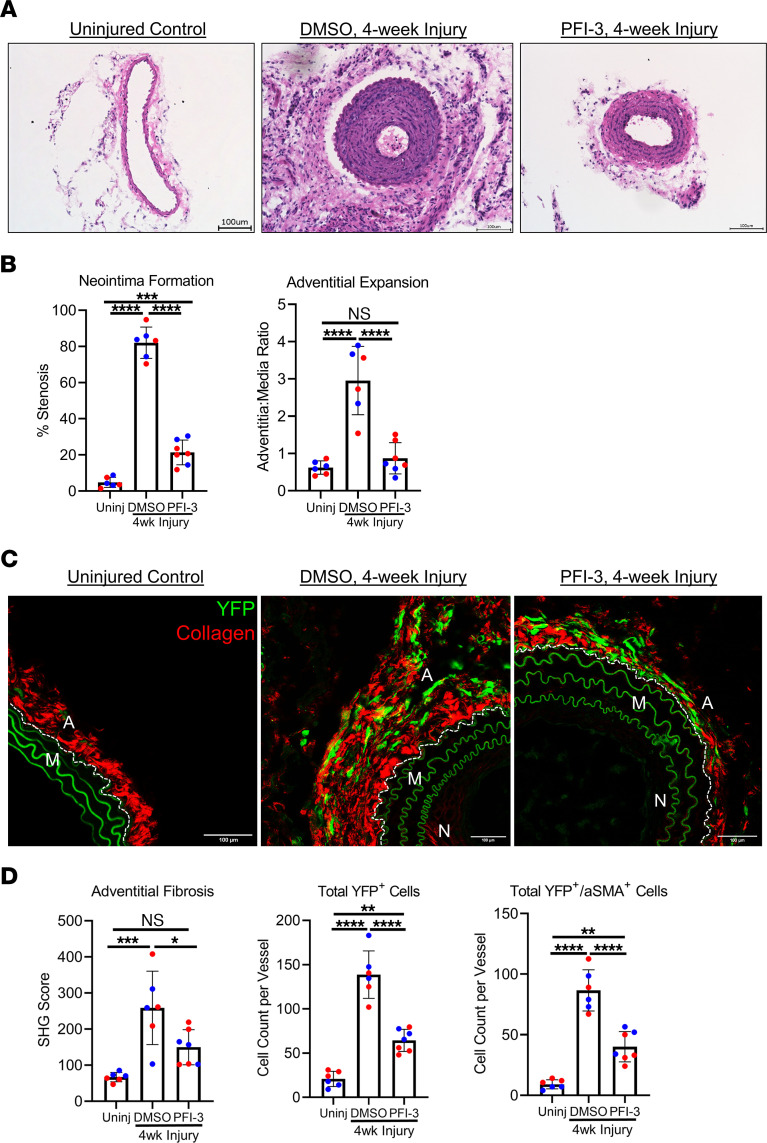
Inhibition of Brg1 bromodomain with PFI-3 attenuates injury-induced pathological vascular remodeling. (**A**) Uninjured and injured carotid arteries were harvested from tamoxifen-treated *Gli1*-Cre^ERT^-YFP AdvSca1-SM reporter mice 4 weeks after ligation, embedded in OCT, and stained with H&E. DMSO mice received 10% DMSO in corn oil by oral gavage every 4 days. PFI-3 experimental mice received 50 mg/kg of PFI-3 by oral gavage every 4 days. Scale bar: 100 μm. (**B**) Percent stenosis was determined by measuring the vessel luminal area and normalizing it to the inner medial circumference, and the adventitia/media ratio was measured by dividing the adventitial area by the outer medial circumference. *n* = 6 (uninjured [Uninj]), *n* = 6 (DMSO), *n* = 7 (PFI-3). Blue data points represent male samples, and red data points represent female samples. A 1-way ANOVA with Tukey’s multiple-comparison test was performed to compare the means of the experimental groups; ***P < 0.001, ****P < 0.0001. (**C**) Uninjured and injured carotid artery sections were harvested 4 weeks after ligation and immunofluorescently stained for YFP (green) to identify AdvSca1-SM–derived cells; they were then imaged for coexpression of label-free SHG for collagen deposition (red). A, Adventitia; M, Media; N, Neointima. Elastin autofluorescence is observed on the green channel. Scale bar: 100 μm. (**D**) Perivascular collagen deposition was measured by dividing SHG integrated density by the outer media circumference. In separate tissue sections, tissues were stained for YFP and αSMA, and total YFP^+^ and YFP^+^/αSMA^+^ cells were counted from stained slides from each group. *n* = 6 (uninjured [Uninj]), *n* = 6 (DMSO), *n* = 7 (PFI-3). Blue data points represent male samples, and red data points represent female samples. A 1-way ANOVA with Tukey’s multiple-comparison test was performed to compare the means of the experimental groups; *P < 0.05, **P < 0.01, ***P < 0.001, ****P < 0.0001.

To probe the significance of Brg1 induction after carotid ligation, we conducted in vivo experiments using Gli1-Cre^ERT2^ ROSA-YFP AdvSca1-SM reporter mice to test the effect of pharmacologically inhibiting Brg1 on ligation-induced pathological vascular remodeling. Tamoxifen-treated AdvSca1-SM reporter mice underwent carotid ligation followed by delivery of either vehicle control (DMSO) or the Brg1 bromodomain inhibitor, PFI-3. PFI-3 is a small molecule validated by x-ray crystallography and computational profiling to have strong specificity for the C-terminal bromodomain of Brg1 and was previously shown to impair stem cell maintenance (26, 27). Uninjured and injured carotid arteries were harvested 4 weeks after ligation and analyzed histologically by H&E staining (Figure 2A). As compared with injured specimens from the control mice that received DMSO, injured carotid arteries from mice that received PFI-3 (50 mg/kg, a dose that has been previously shown to elicit in vivo effects at this concentration; ref. 28) demonstrated a 60% decrease in neointima area and a 75% reduction in adventitial expansion (Figure 2B). Separate sections were used for label-free second harmonic imaging to measure fibrillar collagen deposition (Figure 2C). As compared with injured specimens from the control mice — which demonstrated robust perivascular collagen with YFP^+^ cells embedded within the collagen matrix, consistent with our previous findings (9) — injured carotid arteries from mice treated with PFI-3 showed diminished perivascular collagen deposition, fewer YFP^+^ AdvSca1-SM–derived cells, and nearly a 50% reduction in the number of YFP^+^ AdvSca1-SM–derived cells that coexpressed αSMA as a marker of an activated myofibroblast (Figure 2D and Supplemental Figure 1; supplemental material available online with this article; https://doi.org/10.1172/jci.insight.164862DS1). The pharmacologic activity of PFI-3 was validated using time-resolved fluorescence energy transfer cell free assay (Supplemental Figure 2). Mice that received PFI-3 did not display behavioral signs of toxicity and were weight stable (Supplemental Figure 3).

**Figure 3:**
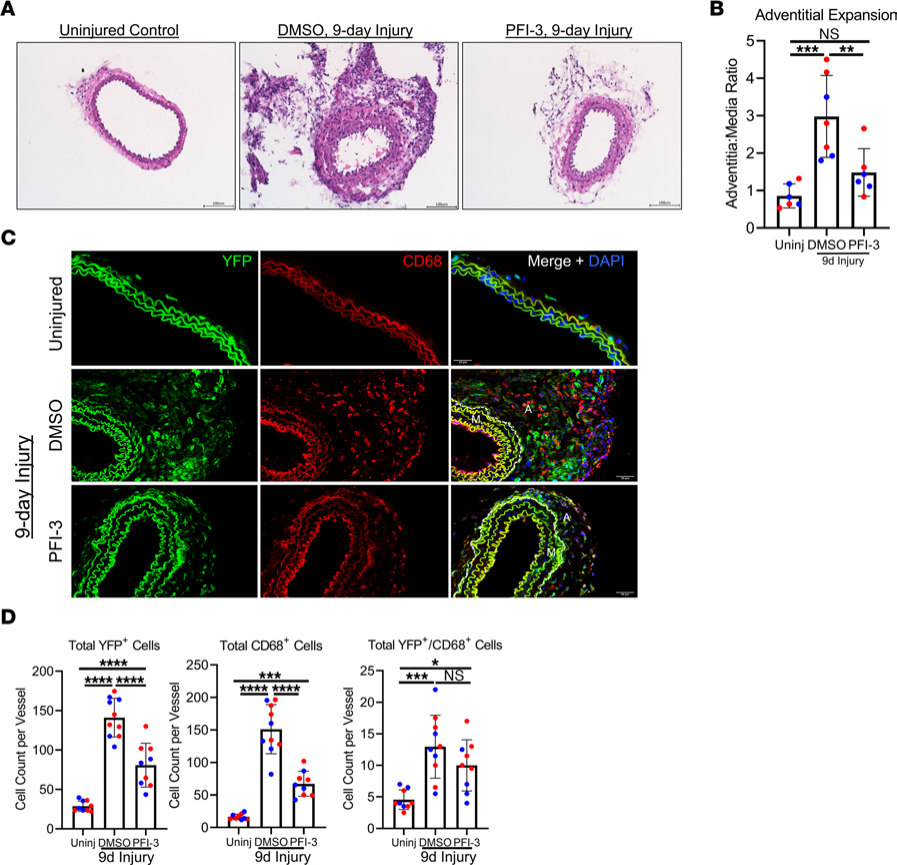
Inhibition of Brg1 bromodomain with PFI-3 decreases macrophage accumulation 9 days after ligation-induced vascular remodeling. (**A**) Uninjured and injured carotid arteries were harvested from tamoxifen-treated *Gli1*-Cre^ERT^-YFP AdvSca1-SM reporter mice 9 days after ligation, embedded in OCT, and stained with H&E. DMSO mice received 10% DMSO in corn oil by oral gavage every 4 days. PFI-3 experimental mice received 50 mg/kg of PFI-3 by oral gavage every 4 days. Scale bar: 100 μm. (**B**) Adventitia/media ratio was measured by dividing the adventitial area by the outer medial circumference. *n* = 6 (uninjured [Uninj]), *n* = 7 (DMSO), *n* = 6 (PFI-3). Blue data points represent male samples, and red data points represent female samples. A 1-way ANOVA with Tukey’s multiple-comparison test was performed to compare the means of the experimental groups; **P < 0.01, ***P < 0.001. (**C**) Uninjured and injured carotid artery sections were harvested 9 days after ligation and immunofluorescently stained for YFP (green) to identify AdvSca1-SM cells and for CD68 (red). “A”=Adventitia, “M”=Media. Elastin autofluorescence is observed on the green and red channels. Scale bar: 50 μm. (**D**) Total YFP^+^ and CD68^+^ cells were counted from stained slides from each group. *n* = 9 (uninjured [Uninj]), *n* = 10 (DMSO), *n* = 9 (PFI-3). Blue data points represent male samples, and red data points represent female samples. A 1-way ANOVA with Tukey’s multiple-comparison test was performed to compare the means of the 2 experimental groups; *P < 0.05, ***P < 0.001, ****P < 0.0001.

Since early-onset vascular inflammation is a major driver of fibrosis and our previous study suggests that AdvSca1-SM cells produce inflammatory chemoattractants, we next examined the effect of Brg1 inhibition on ligation-induced vascular remodeling at an earlier time point (29). Carotid ligation was performed on tamoxifen-treated Gli1-Cre^ERT2^ ROSA-YFP AdvSca1-SM reporter mice; uninjured and injured carotid arteries were harvested 9 days after ligation and were analyzed histologically by H&E staining (Figure 3A). Injured carotid arteries from mice that received PFI-3 (50 mg/kg) showed decreased adventitial expansion as compared with injured arteries from mice that received DMSO (Figure 3B). Uninjured and injured arteries were immunofluorescently stained to study macrophage infiltration, as defined by expression of CD68 (Figure 3C) (30). Injured specimens from mice treated with DMSO showed a significant accumulation of CD68^+^ macrophages and expansion of YFP^+^ AdvSca1-SM–derived cells. In contrast, injured samples from mice treated with PFI-3 showed nearly a 3-fold reduction in CD68^+^ macrophage accumulation and decreased YFP^+^ AdvSca1-SM–derived cell expansion (Figure 3D). Under defined conditions AdvSca1-SM cells were shown to adopt a macrophage-like phenotype; however, our previous report revealed that, in response to acute vascular injury, few YFP^+^ AdvSca1-SM–derived cells expressed macrophage markers (9). Consistent with this, we found occasional YFP^+^ AdvSca1-SM–derived cells that coexpressed CD68 (Figure 3D), suggesting that AdvSca1-SM cells can differentiate toward a macrophage-like phenotype to contribute to inflammatory processes during early stages of vascular remodeling. Collectively, these findings suggest that Brg1 activity underlies cellular changes that drive pathological vascular remodeling.

### Perivascular shRNA-mediated Brg1 knockdown reduces injury-induced pathological vascular remodeling.

**Figure 4:**
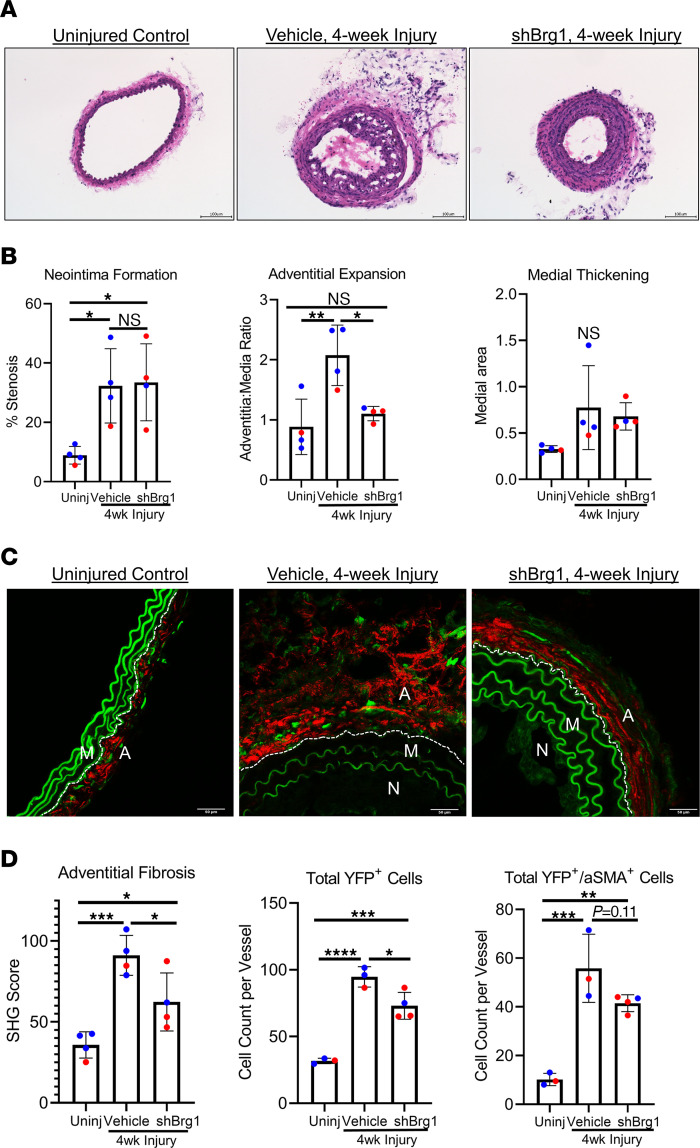
Perivascular knockdown of Brg1 by lentivirus transduction of *Smarca4* shRNA decreases ligation-induced adventitial expansion and fibrosis. (**A**) Uninjured and injured carotid arteries were harvested from tamoxifen-treated *Gli1*-Cre^ERT^-YFP AdvSca1-SM reporter mice 4 weeks after ligation, embedded in OCT, and stained with H&E. The ligated carotid arteries of vehicle mice were coated in 60 μL of a 25% pluronic gel solution. The ligated carotid arteries of shBrg1 mice were coated in 60 μL of a 25% pluronic gel solution containing 2 × 10^7^ lentivirus particles harboring an shRNA sequence targeting *Smarca4*. Scale bar: 100 μm. (**B**) Percent stenosis was determined by measuring the vessel luminal area and normalizing it to the inner medial circumference, and the adventitia/media ratio was measured by dividing the adventitial area by the outer medial circumference. *n* = 4 (uninjured [Uninj]), *n* = 4 (Vehicle), *n* = 4 (shBrg1). Blue data points represent male samples, and red data points represent female samples. A 1-way ANOVA with Tukey’s multiple-comparison test was performed to compare the means of the experimental groups; *P < 0.05, **P < 0.001. (**C**) Uninjured and injured carotid artery sections were harvested 4 weeks after ligation and immunofluorescently stained for YFP (green) to identify AdvSca1-SM–derived cells and imaged for coexpression of label-free SHG for collagen deposition (red). A, Adventitia; M, Media; N, Neointima. Elastin autofluorescence is observed on the green channel. Scale bar: 50 μm. (**D**) Perivascular collagen deposition was measured by dividing SHG integrated density by the outer media circumference. In separate tissue sections, tissues were stained for YFP and αSMA, and total YFP^+^ and YFP^+^αSMA^+^ cells were counted from stained slides from each group. *n* = 4 (uninjured [Uninj]), *n* = 4 (Vehicle), *n* = 4 (shBrg1). Blue data points represent male samples, and red data points represent female samples. A 1-way ANOVA with Tukey’s multiple-comparison test was performed to compare the means of the experimental groups; *P < 0.05, **P < 0.01, ***P < 0.001, ****P < 0.0001.

Because PFI-3 treatment was delivered systemically, there is the potential that many cell types were affected, making it difficult to directly assess the role of Brg1 inhibition on the AdvSca1-SM cells after carotid ligation. To test Brg1 function specifically within the adventitial microenvironment, lentivirus particles (2 × 10^7^ PFU) expressing a *Smarca4*/Brg1-specific shRNA construct were directly administered onto the exposed carotid artery adventitia immediately after ligation (31). Control animals received 25% Pluronic gel as vehicle control. Uninjured and injured carotid arteries were harvested 4 weeks after ligation and analyzed histologically by H&E staining (Figure 4A). As compared with injured specimens from the control mice, injured carotid arteries transduced with the shRNA targeting Brg1 demonstrated a 2-fold reduction in adventitial expansion. Neointima formation and medial thickening were not significantly different between the 2 conditions (Figure 4B). Separate sections were used for label-free second harmonic imaging (Figure 4C). As compared with injured specimens from the control mice — which exhibited abundant perivascular collagen matrix with embedded YFP^+^ cells, consistent with our previous findings — injured carotid arteries from mice that received the shRNA Brg1 construct exhibited diminished perivascular collagen deposition, fewer YFP^+^ AdvSca1-SM–derived cells, and fewer YFP^+^ cells that coexpressed αSMA (Figure 4D and Supplemental Figure 4A). Supplemental Figure 4B demonstrates effective shRNA-mediated Brg1 knockdown. In addition, injured arterial sections from *Smarca4* shRNA–treated mice stained for GFP and Brg1 demonstrated reduced overall adventitial Brg1 expression compared with controls (Supplemental Figure 4C). These findings strengthen the hypothesis that Brg1 facilitates profibrotic changes in AdvSca1-SM cells surrounding the vascular wall after acute vascular injury.

### Inhibition of Brg1 bromodomain blunts TGF-β–induced myofibroblast differentiation of AdvSca1-SM cells in vitro.

**Figure 5:**
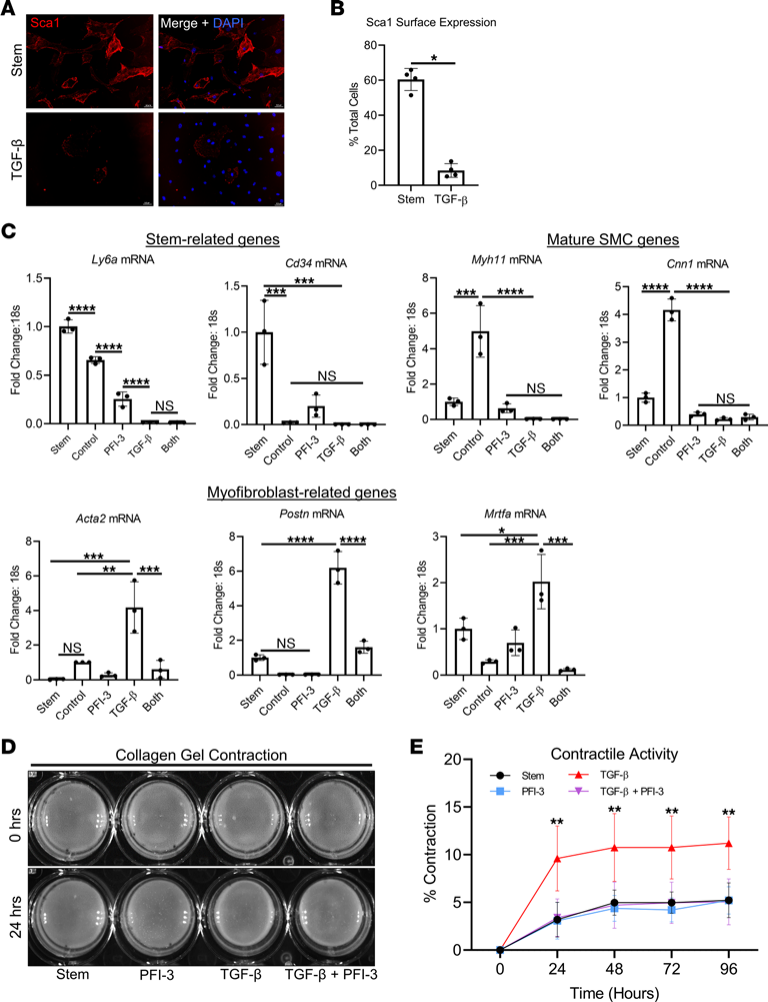
AdvSca1-SM cells stimulated with TGF-β_1_ express myofibroblast-related genes and show enhanced contractility, while PFI-3 blocks TGF-β_1_–mediated myofibroblast gene expression and contractility. (**A**) Subcultured AdvSca1-SM cells isolated from SM22α-Cre-YFP mice were plated onto gelatin-coated glass chamber well slides and were cultured in stem cell media or stimulated with 5 ng/mL TGF-β_1_ for 72 hours. After 72 hours, cells were fixed with 4% PFA for 15 minutes, washed with 1× PBS, and then immunofluorescently stained for Sca1 (red). (**B**) Sca1^+^ cells were counted from stained slides from each group. Four independent samples were measured, and within each sample, 4 images were acquired across the sample to increase coverage of the sample. *n* = 4 (Stem), *n* = 4 (TGF-β). A 2-tailed Mann-Whitney *U* test was performed to compare the medians of the 2 experimental groups; *P < 0.05. (**C**) AdvSca1-SM cells were cultured in stem cell media or high serum media (control) or stimulated with 5 ng/mL TGF-β_1_, 50 μM PFI-3, or TGF-β_1_ + PFI-3 (“Both”) for 72 hours. RNA was harvested for qPCR analysis. Data represent 3 independent experiments. A 1-way ANOVA with Tukey’s multiple-comparison test was performed to compare the means of the experimental groups; *P < 0.05, **P < 0.01, ***P < 0.001, ****P < 0.0001. (**D**) In total, 50,000 AdvSca1-SM cells were seeded onto compressible collagen matrix gels in 24-well plates and cultured in stem cell media or stimulated with 5 ng/mL TGF-β_1_, 50 μM PFI-3, or TGF-β_1_ + PFI-3. Images of collagen matrices were taken every 24 hours. (**E**) Gel area for each well was determined using ImageJ, and data are reported as percent contraction. Four independent replicates were measured. Two-way repeated-measures ANOVA was performed to compare the experimental groups; **P < 0.01.

Given the results obtained from the in vivo studies, we next aimed to functionally and mechanistically study Brg1 regulation of AdvSca1-SM cells using an established AdvSca1-SM cell culture system. When grown under basal conditions with stem cell media, AdvSca1-SM cells retain mRNA expression of *Ly6a* (Sca1) and *Cd34* and retain surface Sca1 protein expression, consistent with their in vivo profile (Figure 5, A and C) (3, 10, 32). To model profibrotic changes observed in AdvSca1-SM cells after vascular injury, cultured AdvSca1-SM cells were stimulated with TGF-β_1_, a well-studied cytokine used to induce myofibroblast differentiation in vascular, cardiac, and pulmonary cell types (33–35). In our system, AdvSca1-SM cells treated with 5 ng/mL TGF-β_1_ led to complete downregulation of the stemness-related genes, *Ly6a* and *Cd34*, along with significant upregulation of the myofibroblast-related genes, *Acta2* and *Postn*, and the myofibroblast-associated transcription factor *Mrtfa* (Figure 5C). TGF-β_1_ stimulation eliminated Sca1 surface expression, indicating loss of the stem phenotype (Figure 5B). AdvSca1-SM cells cultured in high serum-containing media without TGF-β_1_ (“Control”) also led to downregulation of stemness-related genes but, consistent with previous reports, preferential upregulation of genes that define mature SMCs, including *Myh11*/SMMHC and *Cnn1*/Calponin (Figure 5C). These data are consistent with earlier work showing that 50% of AdvSca1 cells grown in high-serum media differentiated into SMCs, while 25% retain Sca1 expression (32). Interestingly, unlike serum stimulation, stimulation with 5 ng/mL TGF-β_1_ blocked the induction of *Myh11* and *Cnn1* mRNA and blocked serum-induced SMMHC protein expression (Figure 5C and Supplemental Figure 5). These results from our in vitro system indicate that TGF-β_1_ blocks AdvSca1-SM cell differentiation into SMCs, while promoting myofibroblast differentiation. We next tested the effect of PFI-3–mediated (50 μM) Brg1 bromodomain inhibition in TGF-β_1_–treated AdvSca1-SM cells. Compared with cells stimulated with TGF-β_1_ alone, cells treated with TGF-β_1_ plus PFI-3 (“Both”) showed at least a 3-fold decrease in the induction of *Acta2*, *Postn*, and *Mrtfa* (Figure 5C). The presence of PFI-3 in TGF-β_1_–stimulated cells did not restore expression of stemness genes (e.g., *Ly6a)* or SMC-specific genes (e.g., *Myh11*, *Cnn1*). To test if the observed changes in gene expression using TGF-β_1_ and PFI-3 conferred functional changes, AdvSca1-SM cells were tested using a collagen gel contraction assay (Figure 5D) (36). While AdvSca1-SM cells cultured in basal conditions with stem cell media or PFI-3 alone elicited minimal contraction of the collagen gels, stimulation with TGF-β_1_ led to a significant increase in contraction, consistent with a TGF-β_1_–mediated contractile myofibroblast phenotype. Coadministration of TGF-β_1_ plus PFI-3 diminished collagen contraction to the levels of AdvSca1-SM cells cultured in stem cell media alone (Figure 5E). The results from these in vitro studies using subcultured AdvSca1-SM cells were consistent with separate experiments using primary AdvSca1-SM cells isolated directly from carotid arteries in situ (Supplemental Figure 6). In addition, AdvSca1-SM cells at passage 10 retained expression of Sca1 and remained responsive to TGF-β_1_ stimulation (Supplemental Figure 7). A total of 50 μM PFI-3 did not affect cell viability (Supplemental Figure 8). Taken together, these in vitro results support the hypothesis that Brg1 functionally blocks TGF-β_1_–mediated AdvSca1-SM cell–to–myofibroblast differentiation.

### siRNA-mediated knockdown of Brg1 decreases TGF-β–induced myofibroblast gene expression of AdvSca1-SM cells in vitro.

Given that the pharmacological approach using PFI-3 led to antifibrotic changes in subcultured AdvSca1-SM cells, we aimed to test if genetic knockdown of Brg1 could confer similar results as the PFI-3 studies. Two independent siRNA constructs targeting the Brg1 gene were used to test the effect of knocking down Brg1 on TGF-β_1_–induced gene expression. Subcultured AdvSca1-SM cells were transfected with 100 nM of siRNA targeting the *Smarca4* gene or scramble control siRNA and then stimulated with TGF-β_1_ for 72 hours. Brg1 knockdown was validated by Western blot, demonstrating that *Smarca4*-specific targeting induced a significant decrease in total Brg1 protein and *Smarca4* mRNA expression (Supplemental Figure 9A). TGF-β_1_–treated AdvSca1-SM cells demonstrated an upregulation of Brg1 protein, as compared with AdvSca1-SM cells cultured in basal conditions, that was blocked in the *Smarca4* siRNA–treated cells; this was not observed for *Smarca4* mRNA (Supplemental Figure 9, A–C). Consistent with the PFI-3 studies, TGF-β_1_ led to complete downregulation of the stemness-related gene *Ly6a*, which was not rescued by Brg1 knockdown (Supplemental Figure 9D). Compared with cells stimulated with TGF-β_1_ alone, cells treated with TGF-β_1_ plus siRNA targeting *Smarca4* showed a significant decrease in the induction of *Acta2*, *Postn*, and *Col1a1* (Supplemental Figure 9D). Therefore, the siRNA studies show that Brg1 knockdown blunts TGF-β–induced myofibroblast gene expression (see complete unedited blots in the supplemental material). By observing consistent results using both pharmacologic inhibition with PFI-3 and genetic siRNA-mediated Brg1 knockdown, these data strengthen the hypothesis that Brg1 mediates myofibroblast differentiation of AdvSca1-SM cells.

### Global unbiased RNA-Seq supports that pharmacologic inhibition of Brg1 is associated with antifibrotic changes in the AdvSca1-SM transcriptome.

**Figure 6:**
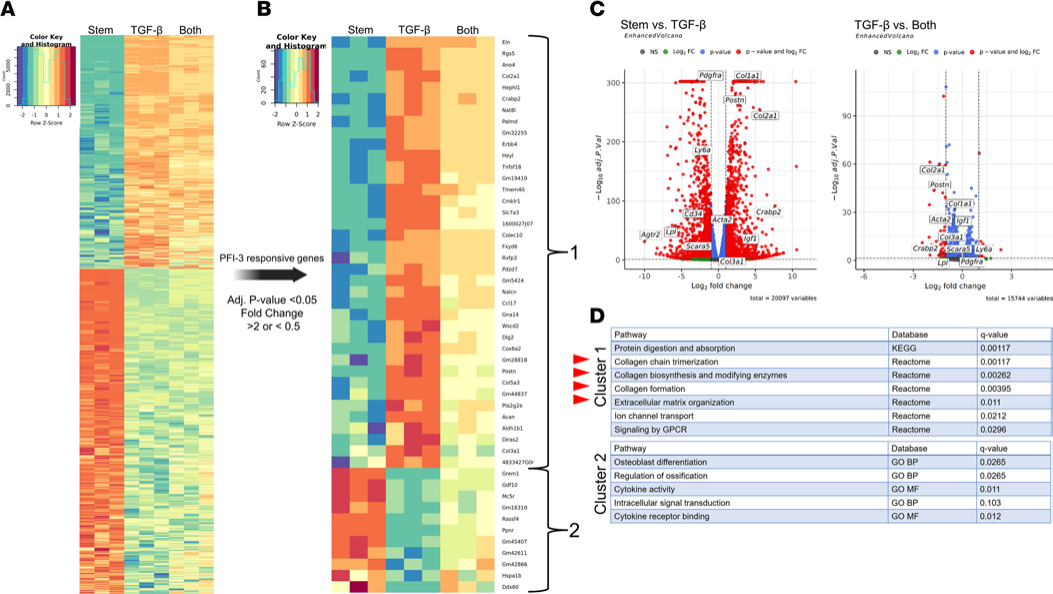
Unbiased RNA-Seq of subcultured AdvSca1-SM cells reveals Brg1 bromodomain inhibition blunts TGF-β_1_–inducible genes related to fibrosis. (**A**) Subcultured AdvSca1-SM cells isolated from SM22α-Cre-YFP mice were cultured in stem cell media or stimulated with 5 ng/mL TGF-β_1_ or 5 ng/mL TGF-β_1_ + 50 μM PFI-3 (“Both”) for 72 hours. RNA was harvested for bulk RNA-Seq. Three independent replicates per condition were included for analysis. The heatmap shows 4,579 differentially expressed genes across experimental groups with the parameters of *P*_adj_ < 0.05 and log_2_ fold change < –1 or > 1. (**B**) The data from the global heatmap were filtered for genes that showed PFI-3 responsiveness with the parameters of *P*_adj_ < 0.05 and log_2_ fold change < –1 or > 1 leading to 49 differentially expressed genes within 2 clusters. (**C**) Volcano plots of the 4,579 differentially expressed genes comparing Stem versus TGF-β and TGF-β versus Both with the parameters of *P*_adj_ < 0.05 and log_2_ fold change < –1 or > 1. (**D**) Gene set overrepresentation analysis of Cluster 1 and Cluster 2 from **B**. Red arrowheads indicate fibrosis- and extracellular matrix–rich pathways.

To expand our understanding of the role of Brg1 on AdvSca1-SM phenotype control, we performed unbiased RNA-Seq to capture global transcriptomic changes in the setting of TGF-β_1_ and PFI-3 stimulation. Subcultured AdvSca1-SM cells were grown in stem cell media to maintain the stem cell phenotype, stimulated with 5 ng/mL of TGF-β_1_ or cotreated with 5 ng/mL TGF-β_1_ plus 50 μM PFI-3 for 72 hours. Total RNA was extracted for amplification, library generation, and sequencing, followed by genome alignment and hierarchical clustering analysis. The global representation of the data revealed 4,579 differentially expressed genes and clear differences in clustering across the experimental conditions, predominantly between the “Stem” and “TGF-β” conditions (Figure 6A). Multidimensional scaling (MDS) analysis of the experimental conditions showed separation of the 3 experimental conditions, with “Stem versus TGF-β” accounting for the majority of separation (Supplemental Figure 10A). To more closely interrogate TGF-β inducible genes that were affected by PFI-3, we filtered all the differentially expressed genes for genes with a TGF-β response that was rescued by PFI-3 (filtering criteria: adjusted *P* [*P*_adj_] < 0.05, log_2_ fold change < –1 or > 1) (Figure 6B). This filtering resulted in 49 differentially expressed genes with 2 major clusters: cluster 1 represents genes upregulated by TGF-β alone and partially reversed with PFI-3 cotreatment and consisted of 38 genes including *Postn*, *Col2a1*, and *Col3a1*. Cluster 2 represents genes downregulated by TGF-β alone and partially reversed with PFI-3 cotreatment and consisted of 11 genes (Figure 6B). Volcano plots of key stemness–related and fibrosis-related genes are shown in Figure 6C. Unbiased gene set overrepresentation analysis using the publicly available resource Consensus Path Database from the Max Planck Institute for Molecular Genetics (http://cpdb.molgen.mpg.de/MCPDB) was applied to cluster 1 and 2 from the heatmap shown in Figure 6B. The database outputted pathway terms for cluster 1 that were associated with extracellular matrix and collagen formation, while cluster 2 was associated with osteoblast formation (Figure 6D). Collectively, these data provide evidence to indicate that inhibition of the Brg1 bromodomain blunts transcriptomic changes associated with TGF-β_1_–mediated AdvSca1-SM–to–myofibroblast differentiation.

### TGF-β_1_ stimulation induces genome-wide increases in Brg1 occupancy of DNA and bromodomain inhibition impedes the recruitment of Brg1 onto fibrosis-related gene promoters.

**Figure 7:**
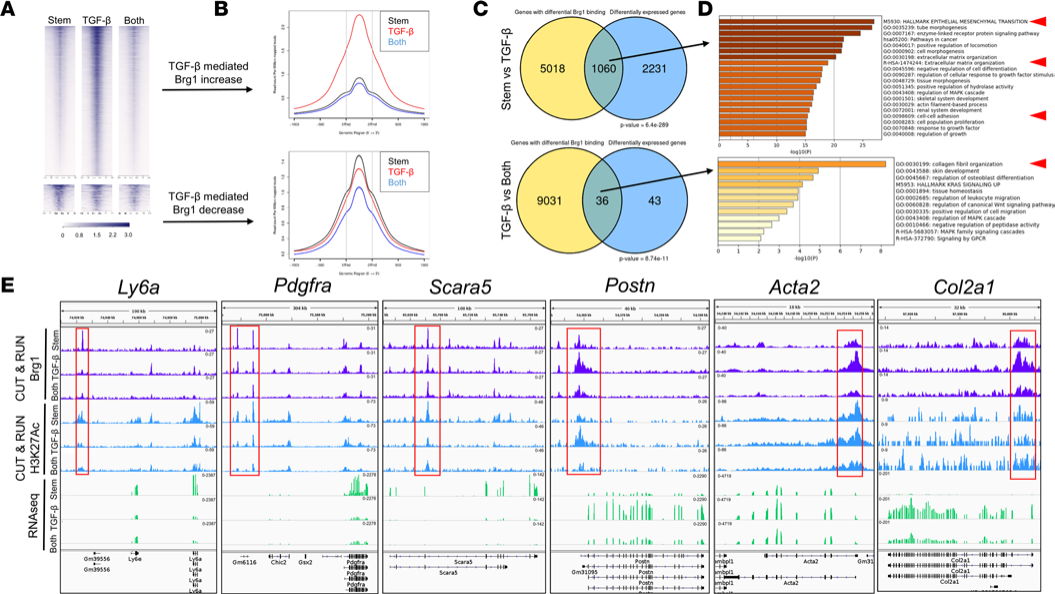
CUT&RUN assay reveals TGF-β_1_–mediated redistribution of Brg1 from distal enhancer sites of stem genes to promoters of myofibroblast genes, while PFI-3 blocks Brg1 occupancy on myofibroblast genes. Subcultured AdvSca1-SM cells isolated from SM22α-Cre-YFP mice were cultured in stem cell media or stimulated with 5 ng/mL TGF-β_1_ or 5 ng/mL TGF-β_1_ + 50μM PFI-3 (“Both”) for 72 hours. Cells were adsorbed to Concanavalin A beads, permeabilized, and incubated with 0.5 μg of anti-Brg1 or anti-H3K27Ac antibody overnight. pAG-MNase was added to the samples to facilitate chromatin digestion and release of enriched DNA bound by Brg1. Enriched DNA was submitted for library preparation and sequenced and aligned to the mouse genome. Peaks represent enriched DNA fragments associated with Brg1 binding. (**A**) Heatmap representing the read intensity at Brg1 peaks across the AdvSca1-SM genome. (**B**) Read count per million mapped reads for all conditions tested shows TGF-β_1_ leads to increased Brg1 peaks on 12,562 genes and decreased Brg1 peaks on 310 genes as compared with AdvSca1-SM cells in the stem condition. (**C**) Comparative analysis of the differential Brg1 binding from CUT&RUN versus differential gene expression from RNA-Seq comparing “Stem” versus “TGF-β” and “TGF-β” versus “Both” with the parameters of *P*_adj_ < 0.05 and 2-fold change. (**D**) The overlapping genes that showed differential Brg1 peak intensity from CUT&RUN and differential gene expression from RNA-Seq were subject to Metascape pathway analysis. (**E**) Representative IGV plots of stemness-related genes (left 3 plots) and myofibroblast-related genes (right 3 plots). Stemness-related genes showed decreased Brg1 reads in distal intergenic regions when stimulated with TGF-β_1_. Myofibroblast-related genes showed increased Brg1 reads near promoter regions when stimulated with TGF-β_1_; PFI-3 blocked the recruitment of Brg1 to promoter regions of myofibroblast genes.

To investigate the mechanism by which Brg1 regulates expression of TGF-β_1_ inducible myofibroblast genes in AdvSca1-SM cells, we conducted “Cleavage Under Target Release Under Nuclease” (CUT&RUN) assays to interrogate Brg1-DNA binding throughout the genome in conditions where cells were maintained in stem cell medium, in cells stimulated with TGF-β_1_, and in cells cotreated with TGF-β_1_ plus PFI-3. CUT&RUN is a technique similar to ChIP-Seq but offers increased specificity because the technique does not require DNA shearing and, instead, takes advantage of micrococcal nuclease to generate highly specific fragments of DNA associated with the protein of interest (37). We hypothesized that stimulation with TGF-β_1_ would lead to Brg1 recruitment to myofibroblast-related gene loci and that inhibiting the Brg1 bromodomain with PFI-3 would disrupt Brg1-DNA binding, thus preventing Brg1-mediated chromatin remodeling and expression of TGF-β_1_–inducible genes. Analysis of all captured reads, which represent all DNA fragments associated with Brg1, revealed that overall, TGF-β_1_ promoted a dramatic increase in Brg1 binding across the AdvSca1-SM genome, and this TGF-β_1_–mediated increase in Brg1 occupancy was nullified in cells treated with TGF-β_1_ plus PFI-3 (Figure 7, A and B). A reduction in Brg1 occupancy was noted in response to TGF-β_1_, although to a much lesser extent, and this was not reversed upon cotreatment with PFI-3 (Figure 7, A and B). To better understand the relationship between Brg1-DNA interactions and subsequent mRNA transcription, we performed comparative analysis to identify the overlapping genes that showed differential Brg1 peaks and differential gene expression among the experimental conditions (Figure 7C). In total, 1,060 overlapping genes were identified in the “Stem versus TGF-β” group, and 36 genes were identified in “TGF-β versus Both” group. Consistent with Brg1 driving an AdvSca1-SM myofibroblast phenotype, pathway analysis on the overlapping genes outputted pathway terms associated with extracellular matrix and collagen formation (Figure 7D). Analysis of individual gene integrative genomics viewer (IGV) plots led to interesting observations. TGF-β_1_ stimulation decreased the amplitude of distal intergenic Brg1 peaks of stemness-related genes, including *Ly6a* and *Pdgfra*, and other genes overrepresented in stemness-like AdvSca1-SM cells, including the scavenger receptor, *Scara5*. (Figure 7E). Since the qPCR and RNA-Seq data showed that TGF-β_1_ induced a strong downregulation of stemness-related gene expression, it is conceivable that the Brg1 peaks that decreased with TGF-β_1_ represent important distal regulatory enhancers for these stemness-related genes, suggesting that Brg1 enrichment at these loci are important for the maintenance of the stem cell phenotype in basal conditions. However, the CUT&RUN studies alone are insufficient to define the function of these distal intergenic peaks. Future studies are warranted to fully characterize these intergenic loci to determine if they are required for the expression of stem-related genes. Additionally, we observed that TGF-β_1_ caused an increased amplitude of major Brg1 peaks near the promoter regions of myofibroblast-related genes, including *Acta2*, *Postn*, and *Col2a1* (Figure 7E). Importantly, AdvSca1-SM cells treated with TGF-β_1_ plus PFI-3 showed decreased amplitude of Brg1 peaks mapped to promoters of myofibroblast genes as compared with AdvSca1-SM cells treated with TGF-β_1_ alone, consistent with the blunting of myofibroblast gene expression we observed in cells treated with TGF-β_1_ plus PFI-3. We also performed CUT&RUN for acetylated histone residue H3K27, a common marker of the open chromatin state. While the relationship was not completely concordant, H3K27Ac peaks predominantly demonstrated increased amplitude at the promoters of fibrosis-related genes in cells stimulated with TGF-β_1_, with the H3K27Ac peaks blunted in the presence of PFI-3 (Figure 7E). This suggests that PFI-3 is sufficient to disrupt the Brg1 bromodomain from binding to target sites and interfere with SWI/SNF-mediated chromatin remodeling necessary for target gene expression. The occupancy of Brg1, as illustrated by the IGV plots, was concordant with the observed changes in mRNA expression captured by the RNA-Seq experiment (Figure 7E and Supplemental Figure 11). However, not all genes upregulated by TGF-β_1_ treatment were necessarily Brg1 dependent. For instance, *Acta2*, *Postn*, *Col2a1*, and *Crabp2*, highly upregulated by TGF-β_1_, are Brg1 dependent as mRNA expression and Brg1 occupancy (and loss of occupancy mediated by PFI-3) were concordant. Other genes upregulated by TGF-β_1_ and exhibiting increased Brg1 occupancy and reduced occupancy with PFI-3 — for instance, *Agtr2* and *Lpl* — appeared to be Brg1 independent as gene expression and Brg1 promoter occupancy were discordant (Supplemental Figure 12). There were no significant differences in Brg1 occupancy on the housekeeping gene *Gapdh* (Supplemental Figure 11). Pathway analysis of the nonoverlapping genes from Figure 7B is illustrated in Supplemental Figure 13. Principle component analysis of the 3 experimental conditions in the CUT&RUN assay reveals strong separation, and genomic enrichment and transcription start site analysis of the 3 experimental groups indicate that there were minor changes in the overall Brg1 genomic localization and distance from the transcription start site (Supplemental Figures 14). Finally, the top 500 differential Brg1 binding sites comparing Stem versus TGF-β were subject to transcription factor motif analysis using the publicly available HOMER database from UCSD (http://homer.ucsd.edu/homer/) to identify enriched Brg1-occupied DNA motifs. This analysis revealed many consensus motifs, including SMAD4 and TCF21, known transcription factors associated with TGF-β signaling and myofibroblast differentiation (Supplemental Figure 15). While these CUT&RUN assays are not sufficient to fully delineate the mechanism by which Brg1 affects AdvSca1-SM differentiation, these experiments are the first reported to our knowledge to shed insight into Brg1-DNA interactions in resident vascular progenitor cells.

## Discussion

Perivascular and tissue fibrosis progression is a consequence of chronic disease commonly observed throughout the body. The activated myofibroblasts that underlie fibrosis are often terminally differentiated; fibrosis progression has not been shown to be significantly reversible in certain systems such as pulmonary fibrosis (38, 39). Therefore, targeting cell populations that demonstrate dynamic differentiation abilities could provide an avenue for mitigating or even reversing vascular fibrosis. Our data raise the question as to whether the myofibroblasts in the acute injury model (derived from AdvSca1-SM cells) are terminally differentiated. The data suggest that targeting the progenitor population results in fewer myofibroblasts; thus, the differentiation to myofibroblasts may be more dynamic, which has important therapeutic implications. AdvSca1-SM cells represent a potentially novel cell population that exhibits dynamic differentiation capabilities as they originate from mature SMCs that are reprogrammed to multipotent progenitor cells that can then differentiate into new SMCs, myofibroblasts, adipocytes, or chondrocytes (8). Sca1 is an important marker for progenitor cells in murine vessels, but the Sca1 gene was evolutionarily deleted between mouse and rat speciation and, thus, is not present in humans (40). However, resident adventitial progenitor cells that express progenitor markers including CD34, SCARA5, and CD90 have been characterized in human vascular tissue and demonstrate multipotent differentiation capabilities (41–43). The existence of human adventitial progenitor–like cells that display dynamic phenotypic differentiation reaffirms that these cells could be an attractive target for manipulating their differentiation as a new treatment approach for patients with progressive vascular fibrosis.

Our previous report demonstrated that AdvSca1-SM cells contribute significantly to vascular fibrosis in the setting of injury-induced as well as angiotensin II–mediated pathological vascular remodeling. Here we have shown that it is possible to mitigate fibrosis progression by blocking the differentiation of AdvSca1-SM cells into myofibroblasts by inhibiting the chromatin remodeler Brg1. The SWI/SNF complex is an epigenetic chromatin remodeling machine that exhibits powerful yet nuanced regulation of gene expression, and the combinatorial assembly of the subunits to form the complex is likely critical for governing the specificity of gene regulation. There are approximately 30 genes in the vertebrate genome that encode for proteins similar to the yeast SWI2/SNF2 ATPases (44), and the SWI/SNF complexes found in vertebrates contain 2 alternative ATPases, Brg1 or Brm, but never both. The diversity of the vertebrate ATPase family implicates that the function of this class of proteins is responsible for broad and yet specific modifications to gene regulation (44). Our previous RNA-Seq data show acute vascular injury induced upregulation of *Smarca4*/Brg1 and, interestingly, a downregulation of *Smarca2*/Brm. These data suggest acute vascular injury induces a potential switch between Brm and Brg1 that is important to poise the SWI/SNF complex for AdvSca1-SM–to–myofibroblast differentiation — a focus of our future studies. The CUT&RUN data indicate that Brg1 is enriched at distal intergenic elements of stemness-related genes in basal conditions, and TGF-β_1_ stimulation causes Brg1 redistribution from these distal regulatory sequences to promoter regions of myofibroblast-related genes and to DNA motifs associated with myofibroblast transcription factors. Determinants of the context-specific distribution of Brg1 may involve upstream signaling pathways that induce the formation of Brg1-containing protein complexes. For example, TGF-β stimulation induces the formation of a protein complex composed of Smad2, the histone acetyltransferase p300, and Brg1 in human keratinocytes; Brg1 is required for Smad2 target gene expression. It is possible that the acetylation of histone tails H3K9 and H3K18 is required for Brg1 bromodomain interaction and targeted chromatin remodeling guided by the Smad2 complex. We observed some discordance between transcript levels and Brg1 enrichment, and this can be due to multiple mechanisms including posttranscriptional regulation from microRNA interference or corepressor functions of Brg1 (45). Taken together, these data underlie the complexity of SWI/SNF chromatin remodeling and suggest that Brg1 is important for maintenance of the AdvSca1-SM cell stemness phenotype at baseline, but redistribution of Brg1 induced by TGF-β_1_ leads to a change in the function of Brg1 to promote myofibroblast differentiation.

We recognize that there are some limitations to this study. While PFI-3 is a validated specific inhibitor of the Brg1 bromodomain, we did not identify a reliable serum marker to validate Brg1 inhibition of the Brg1 bromodomain in vivo. While the SM22 mouse model used to isolate smooth muscle–derived progenitor cells has been validated to have a nearly identical transcriptome as the AdvSca1-SM cells from the Gli1 mouse model, we acknowledge the existence of a rare population of medial SMCs that express Sca1 (46). It is possible that these medial Sca1^+^ SMCs composed a portion of the samples used in these studies.

**Figure 8:**
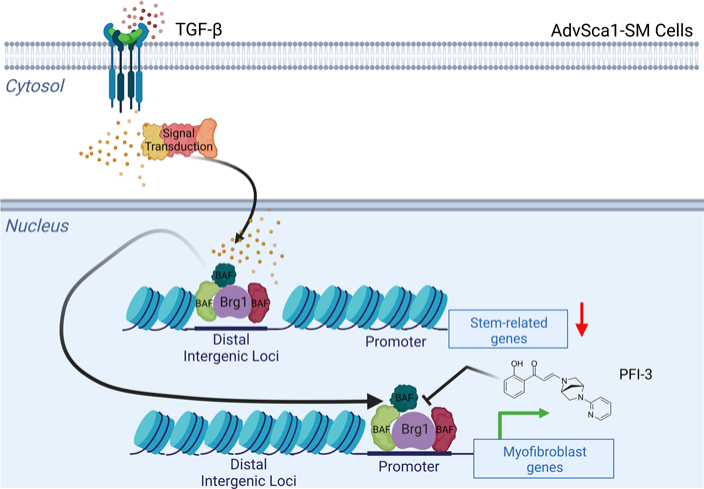
Model illustrating Brg1 facilitating myofibroblast differentiation of AdvSca1-SM cells. Our data suggest that TGF-β_1_ induces Brg1 removal from distal intergenic sites of stemness-related genes and a recruitment to promoters of myofibroblast-related genes. The redistribution of Brg1 occupancy is consistent with the observed changes in gene expression and phenotype observed where AdvSca1-SM cells lose characteristics of the stem phenotype and differentiate into myofibroblasts upon TGF-β_1_ stimulation. The Brg1 bromodomain inhibitor PFI-3 blocks Brg1 recruitment to myofibroblast gene promoters, consistent with PFI-3 inhibiting myofibroblast gene expression.

In conclusion, the model we propose is that Brg1 facilitates differentiation of AdvSca1-SM cells to myofibroblasts by redistributing from intergenic regulatory loci of stemness-related genes to specific gene sets required for myofibroblast differentiation, and Brg1 subsequently remodels chromatin to make the underlying DNA more accessible for transcription factors and other key proteins to carry out gene transcription and, ultimately, phenotypic differentiation (Figure 8). While the translational utility of systemic inhibition of Brg1 by PFI-3 as executed in this study likely also will result in off-target effects, the concept of epigenetic manipulation for human diseases is being applied in the clinic. For example, the DNA methyltransferase inhibitor 5-azacytidine, brand name Vidaza, is an FDA-approved drug used to treat acute myelogenous leukemia (47). Histone deacetylase inhibitors, such as vorinostat, have been developed for human use to treat cutaneous T cell lymphoma (48). As epigenetic drug development has impacted patient outcomes, the translational applications of selective targeting of Brg1 to modify cellular differentiation are realistic and may provide benefit. Therefore, further studies are warranted to increase the translational impact of Brg1 and tissue fibrosis in humans.

## Methods

All supporting information, antibody concentrations, and catalog numbers are detailed and can be found in the Supplemental Methods.

### Animals, carotid artery ligation, PFI-3 treatment, and lentivirus infection.

SM22α-Cre (*TagIn*-Cre; stock no. 004746), *Gli1*-Cre^ERT^ (stock no. 007913), and ROSA26-YFP reporter mice (stock no. 006148) were obtained from The Jackson Laboratory. All mice were fully backcrossed to a C57BL/6 genetic background prior to studies. SM22α-Cre transgenic mice were bred to ROSA26-YFP to generate SMC-selective YFP-expressing reporter mice (SM22α-Cre-YFP). YFP labeling of SM22α-Cre-YFP mice allows for fate mapping of mature SMCs and SMC-derived AdvSca1-SM cells, and subsequent flow-sorting for AdvSca1-SM cells based on YFP^+^ and Sca1^+^ expression was used to isolate AdvSca1-SM cells, as previously established (8, 9). *Gli1*-Cre^ERT^ transgenic mice and ROSA26-YFP reporter mice were bred to generate tamoxifen-inducible AdvSca1-SM–cell specific YFP-expressing reporter mice (*Gli1*-Cre^ERT^-YFP) (9). Adult (8- to 10-week-old) *Gli1*-Cre^ERT^-YFP mice received i.p. injections of 1.5 mg tamoxifen (150 μL injection of 10 mg/mL in corn oil) for 12 consecutive days to induce YFP reporter knockin prior to experiments. YFP labeling of *Gli1*-Cre^ERT^-YFP mice allows for fate mapping of AdvSca1-SM cells in the setting of injury, even if they lose all characteristics of AdvSca1-SM cells (e.g., loss of stem-related genes). The carotid ligation surgery was performed on 8- to 10-week-old mice anesthetized by inhalation of 2% isoflurane. A small incision was made in the midline cervical area, and the left carotid artery was isolated and ligated with 6-0 braided silk, just proximal to the carotid bifurcation. The right carotid artery was uninjured and served as an internal control. The incision was sutured with 5-0 wax-coated braided silk, and the mice were placed on a heating pad to recover postoperatively. Uninjured and injured arteries were harvested at indicated time points and immersed in 4% paraformaldehyde in phosphate-buffered saline (PBS) for 14–18 hours at 4°C for fixation and subsequently submerged in 30% sucrose in 1× PBS for 14–18 hours at 4°C for cryoprotection; they were then embedded in OCT for histological analysis. In separate studies, injured and uninjured carotid arteries were harvested and digested to single-cell suspensions to isolate AdvSca1-SM cells by FACS based on YFP^+^/Sca1^+^ expression. Both male and female mice were used and randomly assigned to experimental groups in all studies. For PFI-3 in vivo studies, PFI-3 was prepared the day of treatment at a concentration of 5 mg/mL in a solution of 10% dimethyl sulfoxide (DMSO) and 90% corn oil and warmed in a water bath and vortexed. Mice received PFI-3 via oral gavage at a dose of 50 mg/kg. The mice were treated 1 day before carotid ligation and then every 4 days following carotid ligation. For lentivirus infection studies, 60 μL of a 25% pluronic gel solution containing 2 × 10^7^ PFU lentiviral particles was pipetted directly around the exposed carotid artery immediately after ligation. The gel was allowed to solidify around the ligated artery before closing the incision. Detailed information about the lentivirus can be found in Supplemental Table 5.

### Isolation of AdvSca1-SM cells from vascular tissue.

The aortic arch, descending aorta, and carotid arteries were harvested from 8-week-old SM22α-Cre-YFP mice after perfusion with heparinized saline (20 U/mL). The vessels were combined and minced with a #10 scalpel blade and were then transferred into 10 mL of Digest Buffer (3.2 mg/mL collagenase II, 0.7 mg/mL elastase, and 0.2 mg/mL soybean trypsin inhibitor in HBSS) and incubated at 37°C for 1 hour. During incubation, the solution was agitated by pipetting every 10 minutes to facilitate digestion. Single-cell suspensions were passed through a 70 μm cell strainer and washed twice with sterile 1× PBS + 0.1%FBS. The resulting single cell suspension was centrifuged at 400*g* for 12 minutes at 4°C, and the resulting pellet was resuspended in 100 μL of sterile 1× PBS + 0.1% FBS and incubated with 3 μL of APC-conjugated rat anti–mouse monoclonal Sca1 antibody for 1 hour at 4°C. Antibody manufacturer, catalog number, and dilution for all antibodies used in this manuscript can be found in Supplemental Table 2. After incubation, the cell suspension was submitted for FACS on a MoFlo XDP100 high-speed cell sorter. Events were gated for singlets and live cells (DAPI^–^); AdvSca1-SM cells were defined as the YFP^+^/APC (Sca1)^+^ population; this gating strategy has been previously established and published for isolation of AdvSca1-SM cells (8, 9).

### Immunofluorescence microscopy, second harmonic generation microscopy, and H&E staining.

Fixed tissues embedded in OCT were sectioned at 6 μm using a Leica CM 3050 S Cryostat, and sections were immediately mounted onto 25 × 75 mm Superfrost Plus Microscope glass slides. For immunofluorescence staining, tissue slides were submerged in deionized water for 10 minutes to remove OCT and were then equilibrated in 1× PBS for 5 minutes. Tissues were permeabilized with 100% methanol for 10 minutes and then incubated in 0.05% Tween-20 in 1× PBS for 5 minutes. Tissues were blocked in 3% horse serum in 1× PBS for 1 hour at room temperature and were sequentially incubated with specific primary antibodies diluted in 3% horse serum in 1× PBS at 4°C overnight. For unconjugated antibodies, antigen/antibody complexes were visualized using Alexa Fluor 488–, Alexa Fluor 568–, or Alexa Fluor 647–coupled secondary antibodies at a dilution of 1:500 in 3% horse serum in 1× PBS for 1 hour at room temperature. Isotype controls included the use of goat, rat, and rabbit IgG at the same dilution and species as the primary antibodies. Antibodies used for immunofluorescence staining are detailed in the Supplemental Table 2. After staining with the secondary antibodies, slides were washed 3 times in 1× PBS and were then mounted with VECTASHIELD medium containing DAPI to detect all cell nuclei. Tissues were imaged using a Keyence BZ-X710 all-in-one fluorescence microscope and analyzed with BZ-X Analyzer software. Histological quantification was performed with FIJI version 2.3.051 with the Cell Counter plugin in a blinded manner by 2 independent investigators. Data were averaged from at least 2 separate tissue sections for each sample. For second harmonic generation acquisition, tissues were imaged with a laser-scanning confocal microscope (LSM 780 spectral, Carl Zeiss) and analyzed using ZEN LE software. The imaging parameters were initially defined empirically using multiple samples to optimize the signal/noise ratio and minimize signal saturation. Acquisition settings were kept constant for all measurements for comparative analysis. Collagen deposition was measured using Integrated Density with FIJI version 2.3.051 and normalized to outer medial circumference. For H&E staining, tissue sections were submitted to the University of Colorado Anschutz Medical Campus Cancer Center Histology Core. Imaging for H&E-stained tissues was performed using the Keyence BZ-X710 microscope, and quantification was performed in a blinded manner by 2 independent investigators using FIJI version 2.3.051. Data were averaged from at least 2 separate tissue sections for each sample.

### Cell culture and in vitro studies.

AdvSca1-SM cells were isolated from the aortic arch, descending aorta, and carotid arteries of pooled SM22α-Cre-YFP mice by FACS as described above and maintained on 0.1% gelatin-coated plates at a density of 80,000 cells per well on a 12-well plate or 200,000 cells per well on a 6-well plate. Cells were maintained in αMEM-GlutaMAX Stem Cell media with 10% mesenchymal stem cell–qualified FBS, 1 ng/mL murine basic fibroblast growth factor, 5 ng/mL murine epidermal growth factor, and 1× penicillin/streptomycin to maintain the stem phenotype (33), and cells were used between P2-P6. Manufacturer information for all reagents used in this manuscript can be found in Supplemental Table 6. To induce SMC differentiation, AdvSca1-SM cells were growth arrested with 0.1% mesenchymal stem cell–qualified FBS αMEM-GlutaMAX Stem Cell media with 1 ng/mL murine basic fibroblast growth factor, 5 ng/mL murine epidermal growth factor, and 1× penicillin/streptomycin for 24 hours; then, the media were replaced with DMEM with 10% FBS, 1× L-glutamine (2 mmol/L), and 1× penicillin/streptomycin. To induce myofibroblast differentiation, AdvSca1-SM cells were growth arrested with 0.1% mesenchymal stem cell–qualified FBS αMEM-GlutaMAX Stem Cell media with 1 ng/mL murine basic fibroblast growth factor, 5 ng/mL murine epidermal growth factor, and 1× penicillin/streptomycin for 24 hours; then, the media were replaced with DMEM with 0.1% FBS, 1× L-glutamine, and 1× penicillin/streptomycin before being stimulated with TGF-β_1_ at a concentration of 5 ng/mL. The recombinant TGF-β_1_ was initially reconstituted at 50 μg/mL in an aqueous vehicle containing 4 mM HCl and 0.1% BSA, as per the manufacturer’s recommendation, and aliquoted. To each aliquot of the stock solution immediately prior to use, the 50 μg/mL TGF-β_1_ stock was diluted 1:10 with serum-free DMEM to yield a 5 μg/mL working solution. AdvSca1-SM cells were directly treated with this 5 μg/mL working concentration at a 1:1,000 dilution to yield a final treatment concentration of 5 ng/mL. For cells treated with PFI-3, AdvSca1-SM cells were growth arrested with αMEM-GlutaMAX Stem Cell media with 0.1% mesenchymal stem cell–qualified FBS with 1 ng/mL murine basic fibroblast growth factor, 5 ng/mL murine epidermal growth factor, and 1× penicillin/streptomycin for 24 hours; then, the media were replaced with DMEM with 0.1% FBS, 1× L-glutamine, and 1× penicillin/streptomycin before being treated with PFI-3 at a final concentration of 50 μM. PFI-3 was initially reconstituted in 100% DMSO to a concentration of 50 mg/mL and aliquoted. Immediately prior to use, the stock aliquots were diluted to a working concentration of 50 mM PFI-3 in 100% DMSO. To prepare the final concentration of 50 μM PFI-3, the working concentration (50 mM PFI-3 in 100% DMSO) was added to a conical tube containing DMEM with 0.1% FBS, 1× L-glutamine, and 1× penicillin/streptomycin at a 1:1,000 dilution to yield a final treatment concentration of 50 μM PFI-3 and a final DMSO concentration of 0.1%. The media containing the PFI-3 were gently agitated prior to cell treatment, as PFI-3 initially precipitates in aqueous solutions. DMSO was added to all wells at 0.1% concentration to control for DMSO effects. All cells were grown in a Thermo Forma Series II Water Jacketed CO_2_ (Thermo Fisher Scientific) incubator at 37°C with 5% CO_2_.

### RNA extraction and RNA-Seq.

Total RNA was isolated using RLT lysis buffer (Qiagen) supplemented with 1% β-mercaptoethanol. Samples were processed with QIAshredder and RNeasy Plus kits (Qiagen) to isolate total RNA. RNA quality and quantity were analyzed using a NanoDrop 2000 Spectrophotometer (Thermo Fisher Scientific). RNA integrity was confirmed using TapeStation analysis; then, RNA-Seq library preparation and sequencing were conducted at the Genomics and Microarray Core at the University of Colorado Anschutz Medical Campus. Ribo-depleted libraries were constructed using a SMARTer Stranded Total RNA-Seq kit (Clontech) customized with mouse-specific oligonucleotides for rRNA removal. Directional mRNA-Seq of sorted cell populations was conducted using NovaSEQ 6000.

### Bioinformatics analysis.

RNA-Seq reads were obtained using Illumina HiSeq analysis pipeline, and prealignment read quality was performed using FastQC (https://www.bioinformatics.babraham.ac.uk/projects/fastqc/). Fastq files were preprocessed with Flexbar 3.5.0 (49) to trim 12 bases at the left side of reads and to remove adapter sequences. The fastq files were aligned to the Mus musculus GRCm39 reference genome (Ensemble, release 96) using STAR version 2.7.10b (50) with default settings. Differential gene expression analyses were performed with the R package DESeq2 (51). Differentially expressed genes were identified as *P*_adj_ < 0.05 and log_2_ fold change < –1 or > 1. Clustering was performed using heatmap.2 (version 3.1.3). The R package EnhancedVolcano (version 1.14.0) was employed to generate the volcano plot. Differentially expressed gene lists were subjected to gene set overrepresentation analysis (52) enrichment analysis using the publicly available resource Consensus Path Database from the Max Planck Institute for Molecular Genetics with default parameters and *P*_adj_ <0.05. The accession no. for the RNA-Seq data reported in this paper is GSE211423.

### qPCR.

In total, 0.5–1 μg of total RNA extracted using the Qiagen Kit was reverse transcribed to generate cDNA using qScript XLT cDNA SuperMix (Quantabio). qPCR was performed on a CFX96 C1000 Touch Thermocycler, and the data were analyzed with CFX Maestro Software (Version 4.1.2433.1219, Bio-Rad). The thermocycling conditions are: (a) 95°C for 10 minutes, (b) 95°C for 15 seconds, (c) 60°C for 60 seconds, (d) plate read, (e) repeat steps b–d for 39 cycles, (f) 65°C for 30 seconds, and (g) hold at 4°C. 18s ribosomal RNA was used as the reference gene to normalize the data. Sequence-specific primers are listed in Supplemental Table 3.

### CUT&RUN.

The CUT&RUN assay was performed using the CUTANA CUT&RUN Kit from Epicypher, and the Epicypher protocol was followed. Briefly, 800,000 cells per sample were trypsinized with 0.25% trypsin and washed 3 times in wash buffer containing 20 mM HEPES (pH 7.5), 150 mM NaCl, and 0.5 mM Spermidine supplemented with protease inhibitor cocktail. The cells were then adsorbed to Concanavalin A beads for 20 minutes at room temperature. Cells were then permeabilized with Antibody Buffer containing 0.1% digitonin and 2 mM EDTA. Samples were incubated with 0.5 μg of anti-Brg1, anti-H3K4me3, or IgG antibody overnight at 4°C. The following day, cells were washed twice in wash buffer plus 0.1% digitonin and incubated with pAG-micrococcal nuclease (pAG-MNase) for 20 minutes at room temperature; then, cells were incubated with 1 mM CaCl_2_ for 2 hours at 4°C to activate pAG-MNase digestion of chromatin fragments under target. The digest reaction was quenched using EpiCypher Stop Buffer, and samples were incubated at 37°C for 30 minutes to liberate digested DNA. The enriched DNA was purified using the EpiCypher DNA column purification kit and quantified using a Quantus fluorometer (Promega). Libraries were prepared with the Tecan/Nugen Ultralow DNA Input Kit with 14 cycles of amplification, and library distribution was evaluated using TapeStation. The libraries were sequenced on the NovaSeq 6000. The quality of the fastq files was assessed using FastQC and MultiQC (53). Illumina adapters and low-quality reads were filtered out using BBDuk (http://jgi.doe.gov/data-and-tools/bb-tools). Bowtie2 (v.2.3.4.3) (54) was used to align the sequencing reads to the mm10 reference mouse genome. Samtools (v.1.11) (55) was used to select the mapped reads (samtools view -b - q 30) and to sort the bam files. PCR duplicates were removed using Picard MarkDuplicates tool (https://github.com/broadinstitute/picard; commit ID: 62ec81cda59d4434604ee4f9c45d6882cd00c887). The normalization ratio for each sample was calculated by dividing the number of uniquely mapped murine reads of the sample with the lowest number of reads by the number of uniquely mapped murine reads of each sample. These normalization ratios were used to randomly subsample reads to obtain the same number of reads for each sample using samtools view -s. Bedtools genomecov was used to create bedgraph files from the bam files (56). Bigwig files were created using deepTools bamCoverage (57). Peaks were called using MACS2 (v2.1.2) (58). IDR was used to identify the reproducible peaks between the replicates (59). Further processing of the peak data was performed in R, using the following tools: valR (60) and DiffBind (61). Average profiles and heatmaps were generated using ngs.plot (62). IGV tracks for the RNA-Seq data were generated with STAR (50) for the alignment to the mm10 reference genome and deepTools bamCoverage for the generation of the bigwig files. Motif analysis was performed using HOMER (63). Pathway enrichment analysis was performed using Metascape (64). The accession no. for the CUT&RUN data reported in this paper is GSE211423.

### Collagen contraction assay.

Twenty-four–well plates were coated with 2% BSA in sterile 1× PBS for 1 hour prior to adding 500 μL of PureCol EZ Gel solution. The plates were incubated at 37°C for 1 hour until the collagen solidified into compressible matrices. AdvSca1-SM cells suspended in αMEM-GlutaMAX Stem Cell media with 10% mesenchymal stem cell–qualified FBS, 1 ng/mL murine basic fibroblast growth factor, 5 ng/mL murine epidermal growth factor, and 1× penicillin/streptomycin were seeded at 50,000 cells per well on the collagen gels and allowed to adhere overnight. The next day, the cells were growth arrested by serum deprivation in 0.1% mesenchymal stem cell–qualified FBS for 24 hours. The cells were then treated with 5 ng/mL TGF-β_1_ and 50 μM PFI-3, and the gels were immediately detached from the wells using a P200 pipet tip. Well images were captured every 24 hours with Alphaimager Mini system; gel area for each well was determined using FIJI software, and data are reported as percent contraction.

### Statistics.

Data were expressed as mean ± SD. All experiments reported were carried out with at least 3 biological replicates, including both male and female mice. The *n* represents the number of biological replicates and is reported in the corresponding figure legends. Data were analyzed using GraphPad Prism Version 8.3.0 (GraphPad Software). Normality of the data distribution was evaluated using the Shapiro-Wilk test and D’Agostino-Pearson test. Unpaired 2-tailed Student’s *t* test or Mann-Whitney *U* test was performed for comparing between 2 groups. One-way ANOVA with Tukey’s multiple-comparison test or Kruskal-Wallis with Dunn’s multiple-comparison test was used to compare 3 or more groups. For the collagen gel contraction assay, a 2-way repeated measures ANOVA was used to measure the effect of cellular conditions and time on percent contraction. *P* values less than 0.05 were considered statistically significant.

### Study approval.

Mice were maintained in the Center for Comparative Medicine, and procedures were performed under a protocol approved by the IACUC at the University of Colorado Anschutz Medical Campus.

## Author contributions

AJJ and MCMWE designed the studies. AJJ, SL, AMD, KAS, and MFM performed experiments. SL performed all bioinformatics analysis associated with the RNA-Seq data set. EPD performed all bioinformatics analysis associated with the CUT&RUN data set and generated the panels for Figure 7. SL and AMD assisted with mouse surgeries and tissue harvest. MFM served as lab manager for the MCMWE lab. APR helped quantify histology data. AJJ, SL, and MCMWE analyzed and interpreted the experimental data. AJJ and MCMWE wrote the manuscript. SL, AMD, EPD, KSM, RAN, and MWM edited the manuscript.

## Supplementary Material

Supplemental data

## Version 1 03/28/2023

In-Press Preview

## Version 2 05/08/2023

Electronic publication

## References

[1] Majesky MW (2011). The adventitia: a dynamic interface containing resident progenitor cells. Arterioscler Thromb Vasc Biol.

[2] Majesky MW (2012). The adventitia: a progenitor cell niche for the vessel wall. Cells Tissues Organs.

[3] Jolly AJ (2022). Heterogeneous subpopulations of adventitial progenitor cells regulate vascular homeostasis and pathological vascular remodeling. Cardiovasc Res.

[4] Wörsdörfer P (2017). The vascular adventitia: an endogenous, omnipresent source of stem cells in the body. Pharmacol Ther.

[5] Chaldakov GN (2012). Adipoparacrinology – vascular periadventitial adipose tissue (tunica adiposa) as an example. Cell Biol Int.

[6] Tinajero MG, Gotlieb AI (2020). Recent developments in vascular adventitial pathobiology: the dynamic adventitia as a complex regulator of vascular disease. Am J Pathol.

[7] Maiellaro K, Taylor WR (2007). The role of the adventitia in vascular inflammation. Cardiovasc Res.

[8] Majesky MW (2017). Differentiated smooth muscle cells generate a subpopulation of resident vascular progenitor cells in the adventitia regulated by Klf4. Circ Res.

[9] Lu S (2020). Smooth muscle-derived progenitor cell myofibroblast differentiation through KLF4 downregulation promotes arterial remodeling and fibrosis. JCI Insight.

[10] Kramann R (2016). Adventitial MSC-like cells are progenitors of vascular smooth muscle cells and drive vascular calcification in chronic kidney disease. Cell Stem Cell.

[11] Tang J (2020). Arterial Sca1^+^ vascular stem cells generate de novo smooth muscle for artery repair and regeneration. Cell Stem Cell.

[12] Trotter KW, Archer TK (2008). The BRG1 transcriptional coregulator. Nucl Recept Signal.

[13] Reisman DN (2005). The expression of the SWI/SNF ATPase subunits BRG1 and BRM in normal human tissues. Appl Immunohistochem Mol Morphol.

[14] Singh M (2007). Structural ramification for acetyl-lysine recognition by the bromodomain of human BRG1 protein, a central ATPase of the SWI/SNF remodeling complex. Chembiochem.

[15] Trotter KW, Archer TK (2004). Reconstitution of glucocorticoid receptor-dependent transcription in vivo. Mol Cell Biol.

[16] Fryer CJ, Archer TK (1998). Chromatin remodelling by the glucocorticoid receptor requires the BRG1 complex. Nature.

[17] Griffin CT (2008). The chromatin-remodeling enzyme BRG1 plays an essential role in primitive erythropoiesis and vascular development. Development.

[18] Li H (2018). Brg1 promotes liver fibrosis via activation of hepatic stellate cells. Exp Cell Res.

[19] Li Z (2020). Dual roles of chromatin remodeling protein BRG1 in angiotensin II-induced endothelial-mesenchymal transition. Cell Death Dis.

[20] Roy N (2015). Brg1 promotes both tumor-suppressive and oncogenic activities at distinct stages of pancreatic cancer formation. Genes Dev.

[21] Zhou J (2009). The SWI/SNF chromatin remodeling complex regulates myocardin-induced smooth muscle-specific gene expression. Arterioscler Thromb Vasc Biol.

[22] Lino Cardenas CL (2019). HDAC9 complex inhibition improves smooth muscle-dependent stenotic vascular disease. JCI Insight.

[23] Kumar A (1997). Remodeling and neointimal formation in the carotid artery of normal and P-selectin-deficient mice. Circulation.

[24] Kawasaki T (2001). Mouse carotid artery ligation induces platelet-leukocyte-dependent luminal fibrin, required for neointima development. Circ Res.

[25] Moura R (2007). Thrombospondin-1 activates medial smooth muscle cells and triggers neointima formation upon mouse carotid artery ligation. Arterioscler Thromb Vasc Biol.

[26] Fedorov O (2015). Selective targeting of the BRG/PB1 bromodomains impairs embryonic and trophoblast stem cell maintenance. Sci Adv.

[27] Vangamudi B (2015). The SMARCA2/4 ATPase domain surpasses the bromodomain as a drug target in SWI/SNF-mutant cancers: insights from cDNA rescue and PFI-3 inhibitor studies. Cancer Res.

[28] Ding Y (2019). Chromatin remodeling ATPase BRG1 and PTEN are synthetic lethal in prostate cancer. J Clin Invest.

[29] Herring BP (2017). Inflammation and vascular smooth muscle cell dedifferentiation following carotid artery ligation. Physiol Genomics.

[30] Chistiakov DA (2017). CD68/macrosialin: not just a histochemical marker. Lab Invest.

[31] Liu R (2013). Ten-eleven translocation-2 (TET2) is a master regulator of smooth muscle cell plasticity. Circulation.

[32] Passman JN (2008). A sonic hedgehog signaling domain in the arterial adventitia supports resident Sca1+ smooth muscle progenitor cells. Proc Natl Acad Sci U S A.

[33] Kramann R (2015). Perivascular Gli1+ progenitors are key contributors to injury-induced organ fibrosis. Cell Stem Cell.

[34] Wei P (2019). Transforming growth factor (TGF)-β1-induced miR-133a inhibits myofibroblast differentiation and pulmonary fibrosis. Cell Death Dis.

[35] Cho N (2018). Featured article: TGF-β1 dominates extracellular matrix rigidity for inducing differentiation of human cardiac fibroblasts to myofibroblasts. Exp Biol Med (Maywood).

[36] Travers JG (2021). HDAC inhibition reverses preexisting diastolic dysfunction and blocks covert extracellular matrix remodeling. Circulation.

[37] Skene PJ, Henikoff S (2017). An efficient targeted nuclease strategy for high-resolution mapping of DNA binding sites. Elife.

[38] Evans RA (2003). TGF-beta1-mediated fibroblast-myofibroblast terminal differentiation-the role of Smad proteins. Exp Cell Res.

[39] Yu QY, Tang XX (2022). Irreversibility of pulmonary fibrosis. Aging Dis.

[40] Holmes C, Stanford WL (2007). Concise review: stem cell antigen-1: expression, function, and enigma. Stem Cells.

[41] Michelis KC (2018). CD90 identifies adventitial mesenchymal progenitor cells in adult human medium- and large-sized arteries. Stem Cell Reports.

[42] Alessandri G (2001). Human vasculogenesis ex vivo: embryonal aorta as a tool for isolation of endothelial cell progenitors. Lab Invest.

[43] Corselli M (2012). The tunica adventitia of human arteries and veins as a source of mesenchymal stem cells. Stem Cells Dev.

[44] Wu JI (2009). Understanding the words of chromatin regulation. Cell.

[45] Lino Cardenas CL (2018). An HDAC9-MALAT1-BRG1 complex mediates smooth muscle dysfunction in thoracic aortic aneurysm. Nat Commun.

[46] Dobnikar L (2018). Disease-relevant transcriptional signatures identified in individual smooth muscle cells from healthy mouse vessels. Nat Commun.

[47] Dombret H (2015). International phase 3 study of azacitidine versus conventional care regimens in older patients with newly diagnosed AML with >30% blasts. Blood.

[48] Olsen EA (2007). Phase IIb multicenter trial of vorinostat in patients with persistent, progressive, or treatment refractory cutaneous T-cell lymphoma. J Clin Oncol.

[49] Roehr JT (2017). Flexbar 3.0 — SIMD and multicore parallelization. Bioinformatics.

[50] Dobin A (2013). STAR: ultrafast universal RNA-seq aligner. Bioinformatics.

[51] Love MI (2014). Moderated estimation of fold change and dispersion for RNA-seq data with DESeq2. Genome Biol.

[52] Gene Ontology Consortium (2021). The Gene Ontology resource: enriching a GOld mine. Nucleic Acids Res.

[53] Ewels P (2016). MultiQC: summarize analysis results for multiple tools and samples in a single report. Bioinformatics.

[54] Langmead B, Salzberg SL (2012). Fast gapped-read alignment with Bowtie 2. Nat Methods.

[55] Li H (2009). The sequence alignment/map format and SAMtools. Bioinformatics.

[56] Quinlan AR, Hall IM (2010). BEDTools: a flexible suite of utilities for comparing genomic features. Bioinformatics.

[57] Ramírez F (2016). deepTools2: a next generation web server for deep-sequencing data analysis. Nucleic Acids Res.

[58] Zhang Y (2008). Model-based analysis of ChIP-Seq (MACS). Genome Biol.

[59] Qunhua L (2011). Measuring reproducibility of high-throughput experiments. Ann Appl Stat.

[60] Riemondy KA (2017). valr: reproducible genome interval analysis in R. F1000Res.

[61] https://bioconductor.org/packages/devel/bioc/vignettes/DiffBind/inst/doc/DiffBind.pdf.

[62] Shen L (2014). ngs.plot: quick mining and visualization of next-generation sequencing data by integrating genomic databases. BMC Genomics.

[63] Heinz S (2010). Simple combinations of lineage-determining transcription factors prime cis-regulatory elements required for macrophage and B cell identities. Mol Cell.

[64] Zhou Y (2019). Metascape provides a biologist-oriented resource for the analysis of systems-level datasets. Nat Commun.

