# Supplementary material for: Redistribution of the chromatin remodeler Brg1 directs smooth muscle–derived adventitial progenitor–to–myofibroblast differentiation and vascular fibrosis
Source: JCI Insight. 2023 May 8;8(9):e164862. doi: 10.1172/jci.insight.164862 (PMC10243795; doi:10.1172/jci.insight.164862)

SUPPLEMENTAL MATERIALS

Redistribution of the chromatin remodeler Brg1 directs smooth muscle-derived adventitial progenitor-to-myofibroblast differentiation and vascular fibrosis

Austin J. Jolly[1, 2], Sizhao Lu[1,5], Allison M. Dubner[1], Keith A. Strand[1], Marie F. Mutryn[1], Aaron Pilotti-Riley[1], Etienne P. Danis[3], Raphael A. Nemenoff[1,5,6], Karen S. Moulton[4], Mark W. Majesky[7,8], and Mary C.M. Weiser-Evans[1,5,6,9]

[1]Department of Medicine, Division of Renal Diseases and Hypertension, University of Colorado Anschutz Medical Campus, Aurora, CO, USA

[2]Medical Scientist Training Program, University of Colorado School of Medicine, Anschutz Medical Campus, Aurora, CO, USA

[3]Department of Pharmacology, University of Colorado Anschutz Medical Campus, Aurora, CO, USA

[4]Department of Medicine, Division of Cardiology, University of Colorado Anschutz Medical Campus, Aurora, CO, USA

[5]School of Medicine, Consortium for Fibrosis Research and Translation, University of Colorado Anschutz Medical Campus, Aurora, CO, USA

[6]Cardiovascular Pulmonary Research Program, University of Colorado Anschutz Medical Campus, Aurora, CO, USA.

[7]Center for Developmental Biology & Regenerative Medicine, Seattle Children's Research Institute, Seattle, WA 98101

[8]Departments of Pediatrics and Pathology, University of Washington, Seattle, WA, 98195

[9]Corresponding Author:

Mary C.M. Weiser-Evans

Department of Medicine, Division of Renal Diseases and Hypertension

University of Colorado Anschutz Medical Campus

12700 East 19th Avenue, C281

Research Complex 2, Room 7002

Aurora, CO 80045 USA

Email: mary.weiser-evans@cuanschutz.edu

Tel (303) 724-4846

FAX (303) 724-4868

MAJOR RESOURCES TABLE

Table S1: Genetically Modified Animals

	JAX Strain Number	Species	Vendor or Source	Background Strain	Genotype
SM22 SMC Reporter	004746	Mouse	In House Breeding	C57/BL6	SM22α-Cre; Rosa26-YFP
Gli1 AdvSca1-SM Reporter	007913	Mouse	In House Breeding	C57/BL6	*Gli1*-Cre^{ERT2}; Rosa26-YFP

Table S2: Antibodies

Target antigen	Species and Reactivity	Conjugate	Vendor or Source	Catalog Number	Working concentration	Application
Ly-6A/E (Sca-1)	Rat anti-mouse	APC	Thermo Fisher	17-5981-82	1:100	Flow Sorting
Ly-6A/E (Sca-1)	Rat anti-mouse	-	BD Pharmingen	553333	1:100	Immunofluorescence
GFP	Goat anti-mouse	FITC	Abcam	6662	1:200	Immunofluorescence
αSMA	Mouse anti-mouse	Cy3	Sigma-Aldrich	C6198	1:2,000	Immunofluorescence
CD68	Rat anti-mouse	-	Bio-Rad	MCA1957	1:100	Immunofluorescence
Brg1	Rabbit anti-mouse	-	Abcam	110641	1:200	Immunofluorescence
H3K27Ac	Rabbit anti-mouse	-	Abcam	4729	0.5μg	CUT & RUN
Brg1	Rabbit anti-mouse	-	Abcam	110641	1:1,000	Western Blot
Brg1	Rabbit anti-mouse	-	Bethyl	A300-813A	0.5μg	CUT & RUN
β-actin	Mouse ascites fluid, anti-mosue		Sigma	A5441	1:60,000	Western Blot
Goat anti-Rat IgG Secondary Antibody, Alexa Fluor 568nm	Goat-anti Rat	Alexa Fluor 568nm	Thermo Fisher	A-11077	1:500	Immunofluorescence
Goat anti-Rabbit IgG Secondary Antibody, Alexa	Goat anti-Rabbit	Alexa Fluor 568nm	Thermo Fisher	A-11036	1:500	Immunofluorescence

Fluor 568nm						
Rabbit IgG Isotype Control	Rabbit IgG	-	Thermo Fisher	31235	10 µg/mL	Immunofluorescence
Rat IgG Isotype Control	Rat IgG	-	Southern Biotech	0108-01	10 µg/mL	Immunofluorescence
Goat IgG Isotype Control	Goat IgG	-	Thermo Fisher	02-6202	10 µg/mL	Immunofluorescence

Table S3: Primer Sequences for qPCR

Gene	Species	Forward Primer	Reverse Primer
Smarca4	Mouse	5'-CAAAGACAAGCATATCCTAGCCA-3'	5'-CACGTAGTGTGTGTTAAGGACC-3'
Ly6a	Mouse	5'-AGGAGGCAGCAGTTATTGTGG-3'	5'-CGTTGACCTTAGTACCCAGGA-3'
Cd34	Mouse	5'-AAGGCTGGGTGAAGACCCTTA-3'	5'-TGAATGGCCGTTTCTGGAAGT-3'
Myh11	Mouse	5'-AAGCTGCGGCTAGAGGTCA-3'	5'-CCCTCCCTTTGATGGCTGAG-3'
Cnn1	Mouse	5'-AAACAAGAGCGGAGATTTGAGC-3'	5'-TGTCGCAGTGTTCCATGCC-3'
Acta2	Mouse	5'-GTCCCAGACATCAGGGAGTAA-3'	5'-TCGGATACTTCAGCGTCAGGA-3'
Postn	Mouse	5'-TGGTATCAAGGTGCTATCTGCG-3'	5'-AATGCCCAGCGTGCCATAA-3'
Mrtfa	Mouse	5'-AAGGAGGCTATCATTGTGGGC-3'	5'-ACTGACTCGGGACTCCAAGG-3'
Col1a1	Mouse	5'-GACATGTTCAGCTTTGTGGACC-3'	5'-GGACCCTTAGGCCATTGTGTA-3'
18s	Mouse	5'-GCAATTATTCCCCATGAACG-3'	5'-GGCCTCACTAAACCATCCAA-3'

Table S4: siRNA Constructs

siRNA Name	Catalog ID Number	Sequence
Silencer Select siRNA Brg1 #1	s74000	5'-GGCUUGAUGGAACCACAAATT-3'
Silencer Select siRNA Brg1 #2	s73998	5'-GGUCAACGGUGUCCUCAAATT-3'
Negative Control siRNA	AM4611	Proprietary sequence by manufacturer

Table S5: Lentivirus Constructs

Product Name	Vector ID	Catalog Number	Lot Number
Ultra-purified recombinant lentivirus, large-scale packaging, made from pLV[shRNA]-mCherryU6>mSmarca4[shRNA#1]	VB221115-1306mmz	LVLP(VB221115-1306mmz)-K2	221129LVM01
shRNA sequence:	5'-TCGAGTCTCTACCAGCATTAACTCGAGTTAATGCTGGTAGAGACTCGA-3'		

51 **Table S6: Chemicals, Reagents, and Supplies**

Name	Vendor	Catalog Number
Tamoxifen	Sigma-Aldrich	T5648-5G
Isoflurane	Piramal Critical Care	66794-017-25
32% Paraformaldehyde Solution, Methanol-Free	Electron Microscopy Sciences	15714-S
Sucrose	Alfa Aesar	36508
Tissue-Plus™ O.C.T. Compound	Thermo Fisher	23-730-571
Heparin sodium salt from porcine intestinal mucosa	Sigma-Aldrich	H3393
Collagenase Type II	Worthington	LS004177
Elastase Suspension	Worthington	LS002280
Soybean Trypsin Inhibitor	Worthington	LS003571
Fetal Bovine Serum, Standard	Gibco	10437028
Fetal Bovine Serum, Mesenchymal Stem Cell-Qualified	Gibco	12662029
Tween-20	Thermo Fisher	BP337-500
Normal Horse Serum Blocking Solution	Vector Laboratories	S-2000
Bovine Serum Albumin, Heat Shock Treated	Fisher Scientific	BP1600-100
Recombinant Murine Basic Fibroblast Growth Factor	R&D Systems	3139-FB
Murine Epidermal Growth Factor	R&D Systems	2028-EG
VECTASHIELD® Antifade Mounting Medium with DAPI	Vector Laboratories	H-1200
Modified Harris Hematoxylin	Epredia	72711
Xylenes, ACS	LabChem	LC269704
Anhydrous Ethanol, 200 proof	Decon Laboratories	2701
2% Gelatin Solution	Sigma-Aldrich	G1393
MEM α, nucleosides, GlutaMAX™ Supplement	Gibco	32571036
Dulbecco's Modified Eagle Medium	Gibco	11965092
Opti-MEM™ Reduced Serum Medium	Gibco	31985-070
Penicillin-Streptomycin Solution, 100x	Corning	30-002-CI
Penicillin-Streptomycin-L-Glutamine Solution, 100x	Corning	30-009-CI
Trypsin EDTA 1X	Corning	25-053-CI
Recombinant Mouse TGF-β_1 Protein	R&D Systems	7666-MB
PFI-3, powder	SelleckChem	S7315

Dimethyl Sulfoxide	Sigma-Aldrich	D8418
Mammalian Protein Extract Reagent	ThermoScientific	78501
Protease Inhibitor Cocktail	Sigma-Aldrich	P8340
Novex^{TM} WedgeWell 4-12% Tris-Glycine Gel	Invitrogen	XP04125BOX
Lipofectamine^{TM} RNAiMAX Transfection Reagent	Invitrogen	13778-075
PureCol® EZ Gel Neutralized Type I Collagen Solution	Advanced BioMatrix	5074
Braided Silk Suture, 6-0	Teleflex	104-S
Wax Coated Braided Silk Suture, 50	Covidien	S-1173
#10 Scalpel Blades	World Precision Instruments	500239
70µm Nylon Mesh Cell Strainer	Fisherbrand	22363548
Superfrost™ Plus Microscope Slides	Fisherbrand	12-550-15

Table S7: Critical Commercial Assays

Name	Vendor	Catalog Number
QIAshredder	Qiagen	79656
RNeasy Plus Mini Kit	Qiagen	74136
RNeasy Plus Micro Kit	Qiagen	74034
qScript XLT cDNA SuperMix	Quantabio	95161
CUTANA™ ChIC/CUT&RUN Kit	EpiCypher	14-1048
Quantus Fluorometer	Promega	E6150

Supplemental Methods

Western Blot Analysis: AdvSca1-SM were lysed using ice-cold Mammalian Protein Extraction Reagent supplemented with 10 µL/mL protease inhibitor cocktail. Cell lysates were subject to sonication for 5 seconds on ice, then centrifuged at 14,000*g* for 10 minutes at 4°C and supernatant was collected in fresh Eppendorf tubes on ice. 10µg of protein lysate was loaded onto a Novex™ WedgeWell 4-12% Tris-Glycine Gel, separated by SDS-PAGE at 120V, and transferred to polyvinylidene fluoride membranes and blocked for one hour with 5% bovine serum albumin in 1x Tris-buffered saline + 0.1% Tween prior to immunoblotting. β-actin was used as a loading control. Band intensity was quantified using ImageJ and normalized to β-actin expression.

siRNA Transfection: AdvSca1-SM cells were plated at 200,000 cells per well in 6-well dishes and allowed to adhere overnight. The next day, the cells were washed with HBSS, and then transfected with 100nM siRNA constructs diluted in serum free Opti-MEM media and supplemented with Lipofectamine RNAiMAX (4.5µL in 75uL Opti-MEM) and incubated for 6 hours. After the 6-hour incubation, the wells were supplemented with FBS-containing media to bring the total solution to 10% FBS. The cells were incubated for 36 hours at 37°C with 5% CO_2 before experimentation. The siRNA sequences can be found in Supplemental Table S4. The siRNA constructs were purchased from ThermoFisher Scientific.

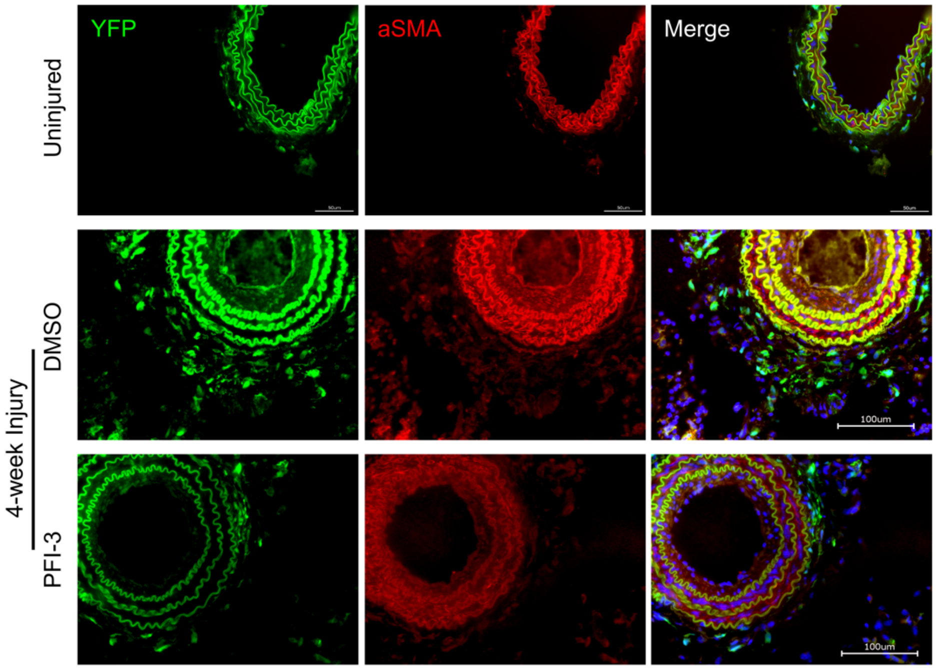

Supplemental Figure I: Immunofluorescent staining of αSMA and YFP four weeks after carotid ligation. Uninjured and injured carotid arteries were harvested from tamoxifen treated Gli1-Cre^{ERT2} ROSA-YFP AdvSca1-SM reporter mice four weeks after ligation, embedded in OCT, and immunofluorescently stained for YFP (green) to identify AdvSca1-SM cells and for αSMA (red). Elastin autofluorescence is observed on the green and red channel. "DMSO" mice received 10% DMSO in corn oil by oral gavage every four days. "PFI-3" experimental mice received 50mg/kg of PFI-3 by oral gavage every four days. Scale bar = 100μm.

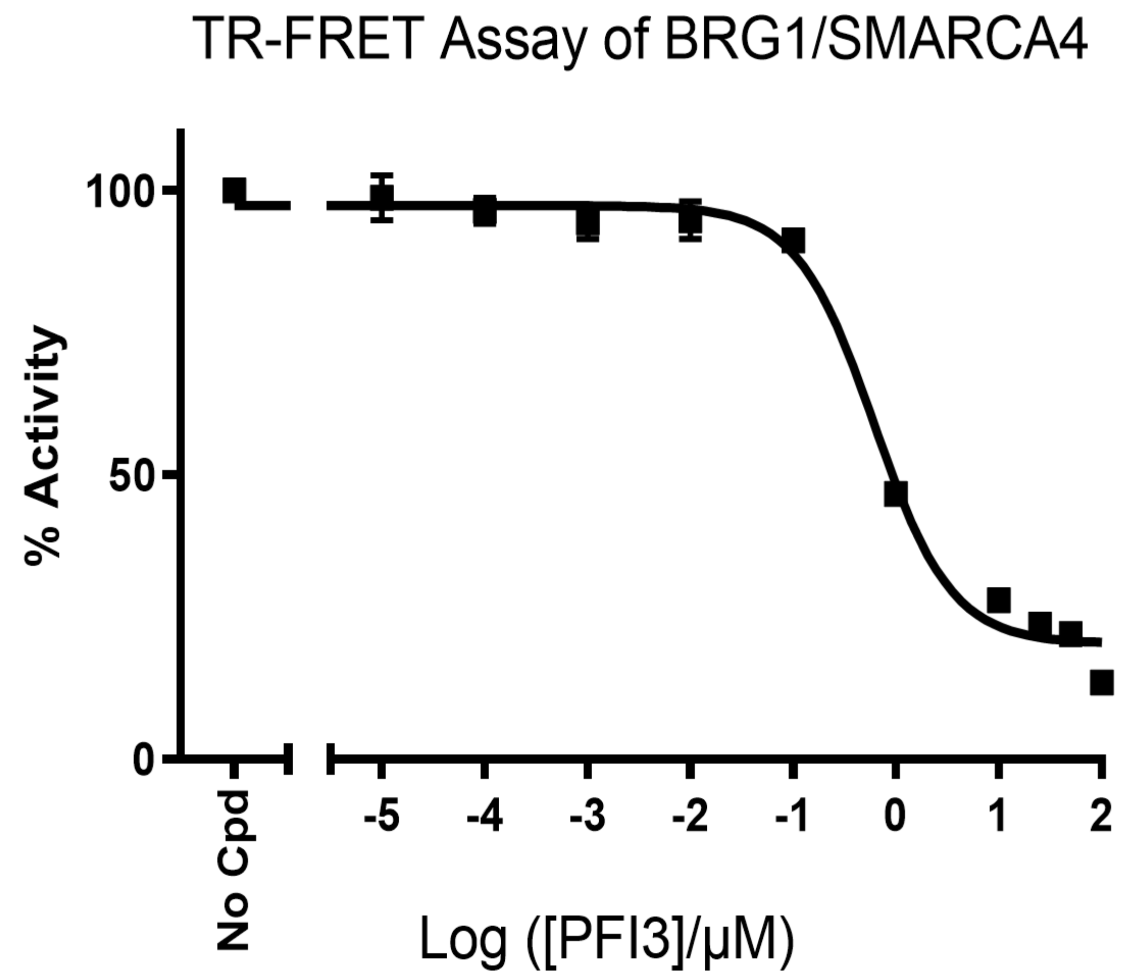

Supplemental Figure II. Validation of PFI-3 activity inhibiting the Brg1 bromodomain. Time Resolved Fluorescence Resonance Energy Transfer (TR-FRET) was used to measure the interaction between purified Brg1 recombinant protein and a peptide derived from histone H3 that possesses an acetylated lysine residue, which is the key residue that binds the Brg1 bromodomain. The TR-FRET signal was measured across various concentrations of PFI-3 to validate the action of PFI-3 to bind the Brg1 bromodomain and inhibit the interaction between Brg1 and the H3 peptide. An aliquot of PFI-3 was submitted to BPS Bioscience for the bromodomain binding assay.

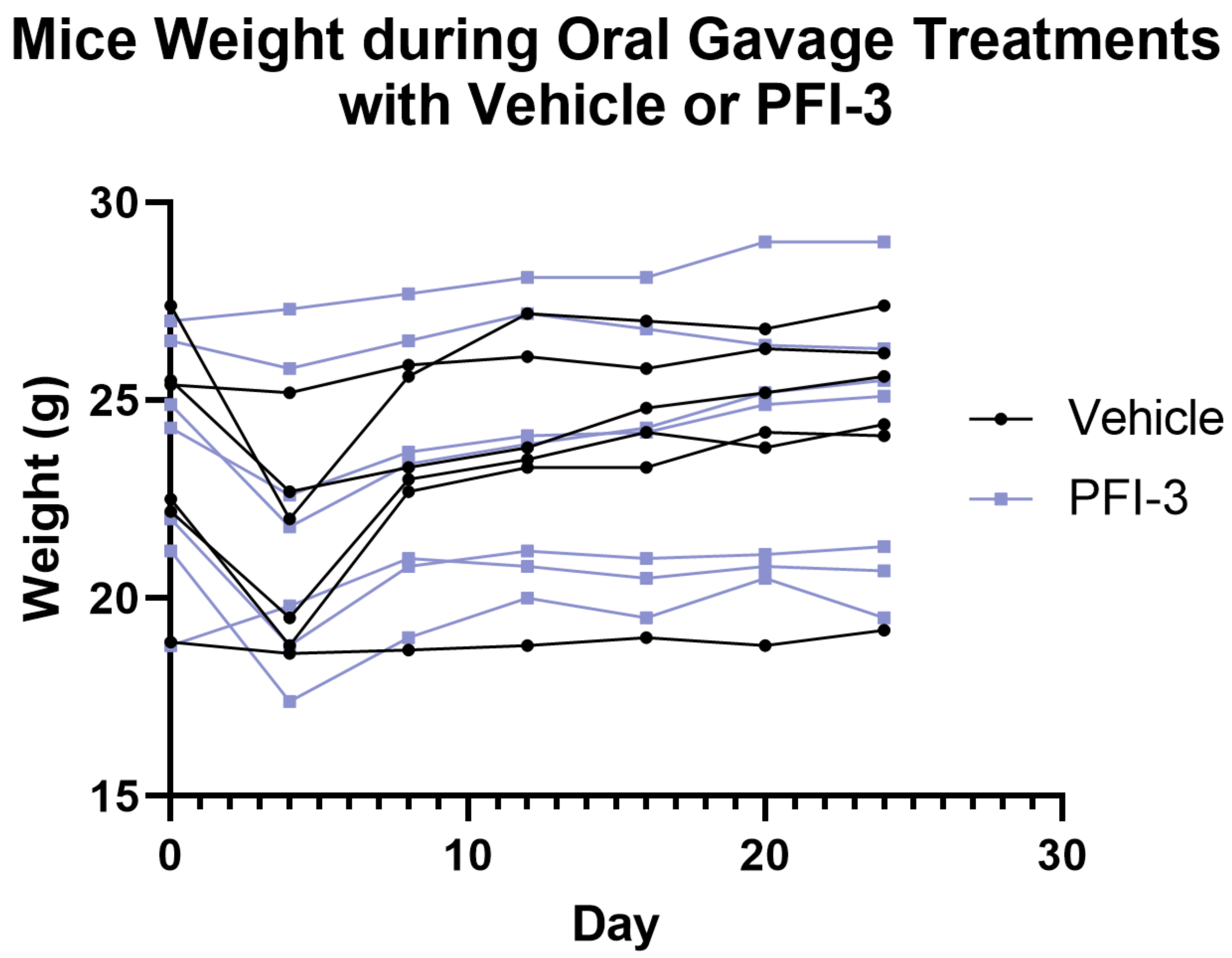

Supplemental Figure III. Mice are weight-stable when treated with PFI-3 over a four-week period. Gli1-Cre^{ERT2} ROSA-YFP mice were administered PFI-3 (50mg/kg) or vehicle (10% DMSO, 90% corn oil) every 4 days for 4 weeks via oral gavage. Weight was recorded the day before carotid ligation surgery and then before each treatment. Over the course of 4 weeks, animals treated with PFI-3 or vehicle maintained stable body weight. The early decrease in weight for some mice can be attributed to the carotid ligation surgery, which involves a recovery period.

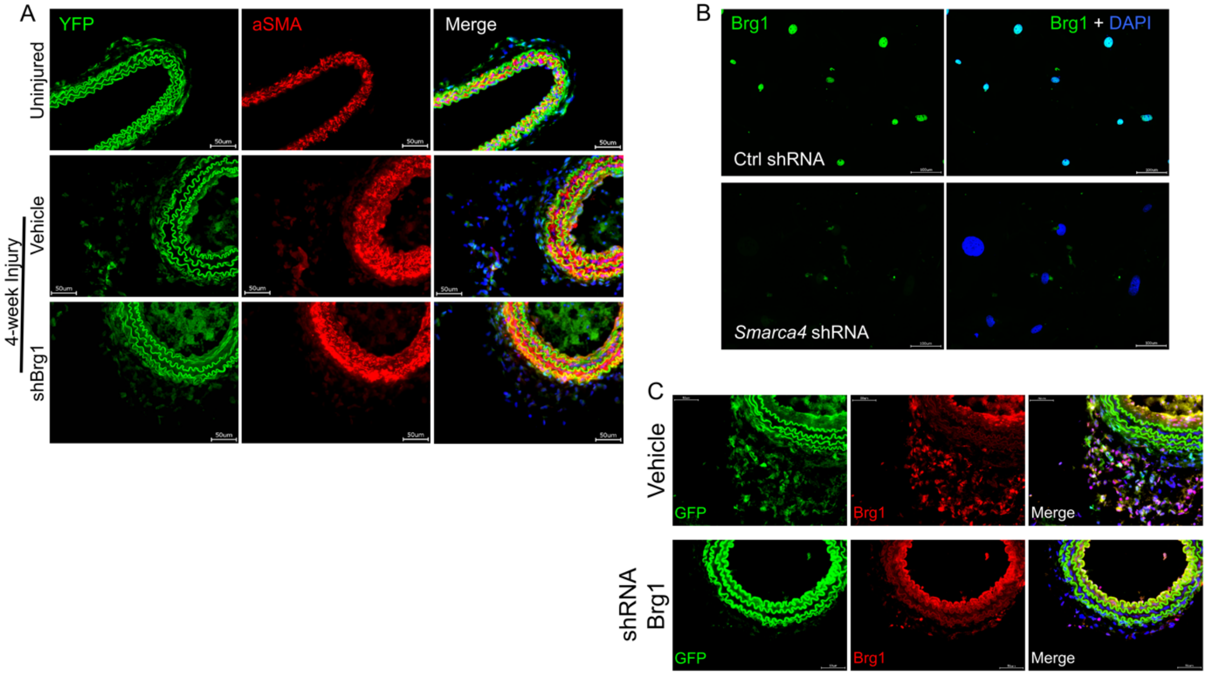

Supplemental Figure IV: Immunofluorescent staining of αSMA and YFP four weeks after carotid ligation with lentivirus and efficient shRNA-mediated Brg1 knockdown. (A). Uninjured and injured carotid arteries were harvested from tamoxifen treated Gli1-Cre^{ERT2} ROSA-YFP AdvSca1-SM reporter mice four weeks after ligation, embedded in OCT, and immunofluorescently stained for YFP (green) to identify AdvSca1-SM cells and for αSMA (red). Elastin autofluorescence is also observed on the green channel. The ligated carotid arteries of "Vehicle" mice were coated in 60μL of a 25% pluronic gel solution. The ligated carotid arteries of "shSmarca4" mice were coated in 60μL of a 25% pluronic gel solution containing 2×10^7 lentivirus particles harboring an shRNA sequence targeting *Smarca4*. Scale bar = 100µm. **(B).** Subcultured AdvSca1-SM cells were treated with control (Ctrl) or *Smarca4* shRNA, fixed, and immunofluorescently stained for Brg1 (green). DAPI (blue) shows all cell nuclei. **(C).** Injured control (vehicle) and *Smarca4* shRNA-treated arteries were stained for YFP (green) to identify AdvSca1-SM cells and Brg1 (red).

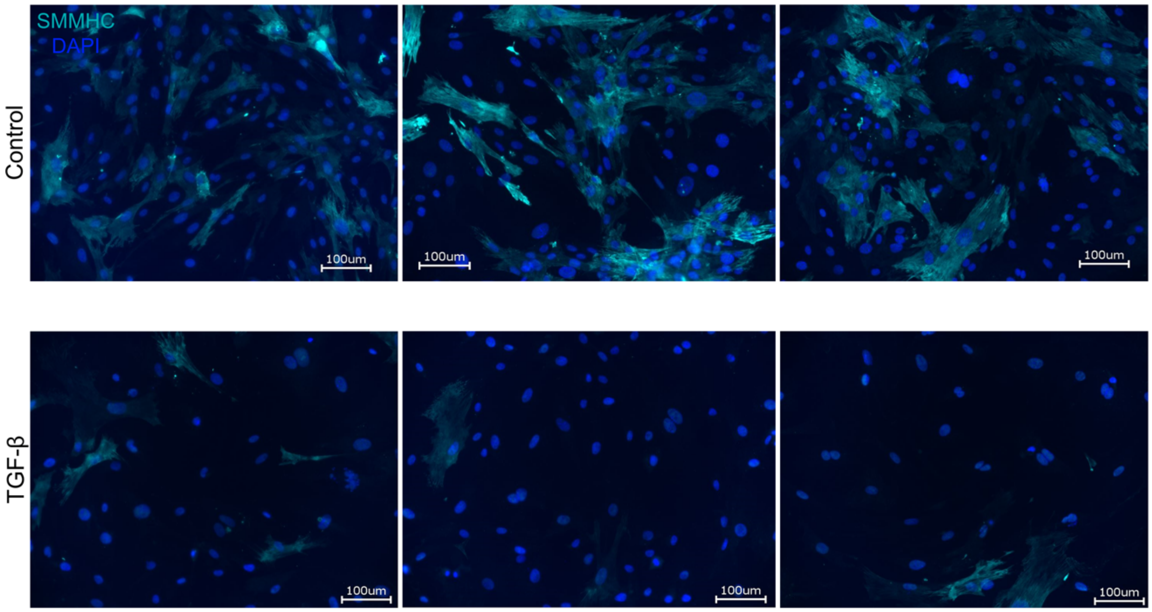

Supplemental Figure V. Primary AdvSca1-SM cells express SMMHC protein in high serum media and TGF-β suppresses protein expression of SMMHC. Freshly isolated AdvSca1-SM cells harvested from carotid arteries and descending aortae of SM22α-Cre ROSA-YFP mice were plated onto gelatin-coated glass chamber well slides and were cultured in high serum DMEM ("Control") or cultured in high serum DMEM and stimulated with 5ng/mL TGF-β_1 for 10 days ("TGF-β"). After 10 days, cells were fixed with 4% PFA for 15 minutes, washed with 1x PBS, then immunofluorescently stained for SMMHC (cyan). Nuclei were stained for DAPI (blue).

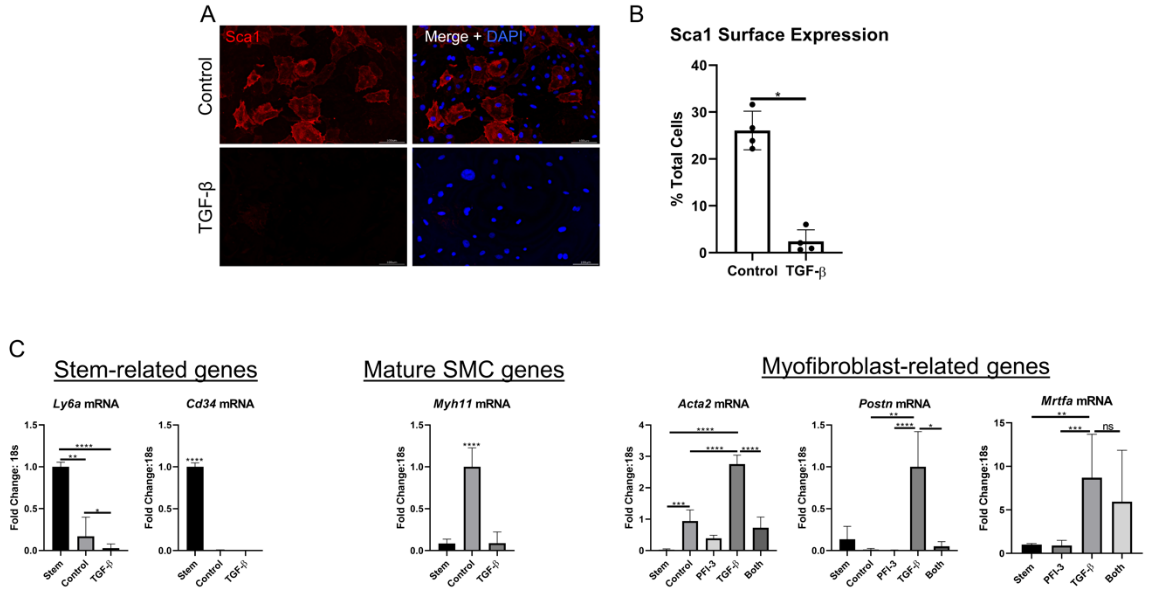

Supplemental Figure VI. Primary AdvSca1-SM cells express SMC related genes in high serum media and TGF-β induces expression of myofibroblast-related genes that is blunted by PFI-3. (A). Freshly isolated AdvSca1-SM cells harvested from carotid arteries and descending aortae of SM22α-Cre ROSA-YFP mice were plated onto gelatin-coated glass chamber well slides and were cultured in high serum DMEM ("Control") or cultured in high serum DMEM and stimulated with 5ng/mL TGF-β_1 for 10 days ("TGF-β"). After 10 days, cells were fixed with 4% PFA for 15 minutes, washed with 1x PBS, then immunofluorescently stained for Sca1 (red). Nuclei were stained for DAPI (blue). **(B).** Sca1$^+$ cells were counted for each group. Four independent samples were measured, and within each sample, four images were acquired to increase coverage of the sample. N=4 (control), N=4 (TGF-β_1). A Mann-Whitney U test was performed to compare the medians of the two experimental groups; *P<0.05. **(C).** Freshly isolated AdvSca1-SM cells from SM22α-Cre ROSA-YFP mice were plated onto 12-well plates and were cultured in high serum DMEM ("Control") or cultured in high serum DMEM and stimulated with 50µM PFI-3 ("PFI-3"), 5ng/mL TGF-β_1 ("TGF-β") or co-stimulated with 5ng/mL TGF-β_1 + 50µM PFI-3 for ten days. RNA was extracted from separate freshly isolated AdvSca1-SM cells to capture AdvSca1-SM cells in their most stem-like state in situ ("Stem"). RNA was harvested for quantitative reverse transcription polymerase chain reaction (qRT-qPCR) analysis. Data represent three independent experiments including technical replicates. A one-way ANOVA with Tukey's Post-Hoc test was performed to compare the means of the experimental groups; *P<0.05, **P<0.01, ***P<0.001, ****P<0.0001.

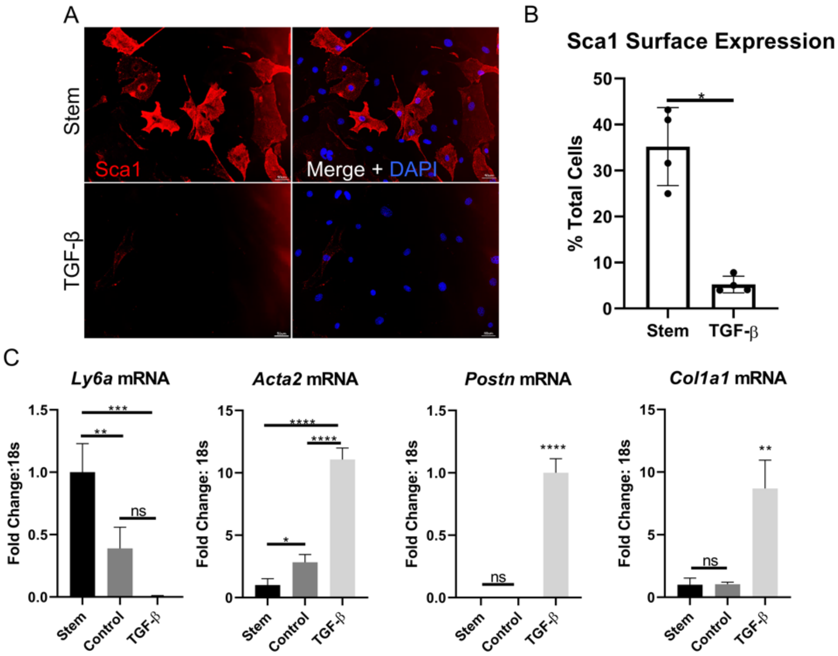

Supplemental Figure VII. AdvSca1-SM cells retain Sca1 expression and are responsive to TGF-β stimulation at passage 10. Subcultured AdvSca1-SM cells isolated from SM22α-Cre ROSA-YFP mice at passage 10 were plated onto gelatin-coated glass chamber well slides and were cultured in stem-cell media supplemented with 0.1% MSC-qualified FBS or cultured in DMEM supplemented with 0.1% FBS and stimulated with 5ng/mL TGF-β_1 for 72 hours. **(A).** After 72hrs, cells were fixed with 4% PFA for 15 minutes, washed with 1x PBS, then immunofluorescently stained for Sca1 (red). Nuclei were stained for DAPI (blue). **(B).** $Sca1^+$ cells were counted for each group. Four independent samples were measured, and within each sample, four images were acquired to increase coverage of the sample. N=4 (Stem), N=4 (TGF-β_1). A Mann-Whitney U test was performed to compare the medians of the two experimental groups; *$P<0.05$. **(C).** Subcultured AdvSca1-SM cells isolated from SM22α-Cre ROSA-YFP mice at passage 10 were cultured in stem-cell media, high serum DMEM media, or stimulated with 5ng/mL TGF-β_1 for 72 hours. RNA was harvested for quantitative reverse transcription polymerase chain reaction (qRT-qPCR) analysis. Data represent three independent biological replicates. A one-way ANOVA with Tukey's Post-Hoc test was performed to compare the means of the experimental groups; *P<0.05, **$P<0.01$, ***$P<0.001$, ****P<0.0001.

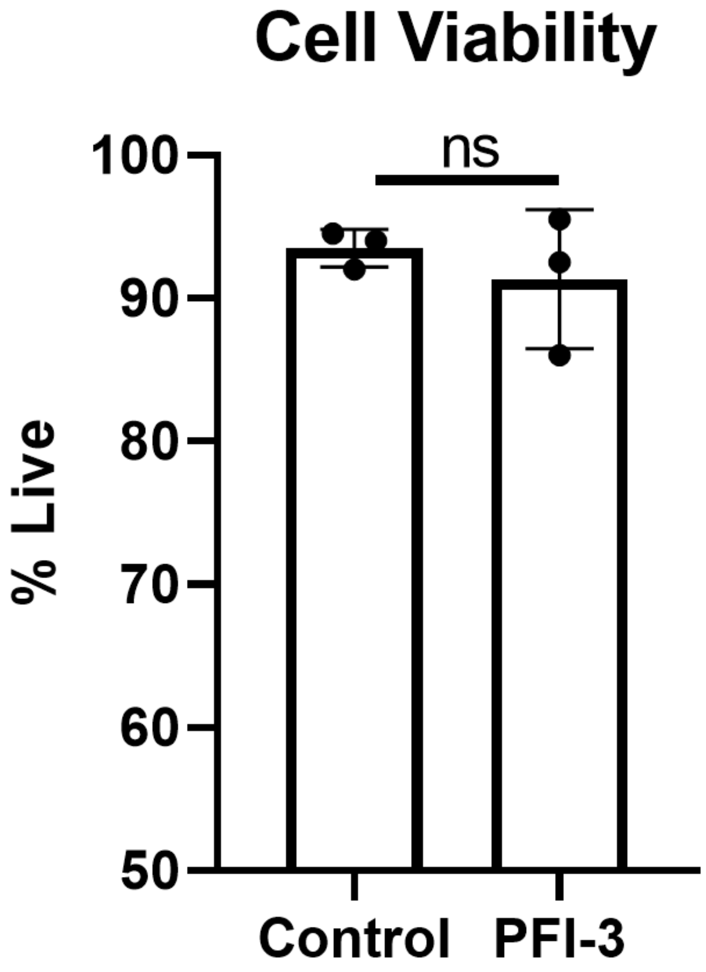

Supplemental Figure VIII. PFI-3 does not decrease AdvSca1-SM cell viability. AdvSca1-SM cells isolated from SM22α-Cre ROSA-YFP mice at passage 4 were plated at 200,000 cells per-well on a gelatin-coated 6-well plate and were incubated for 72hrs in stem-cell media with vehicle (0.1% DMSO, "Control") or PFI-3 (50µM in 0.1% DMSO) for 72 hours. At 72 hours, the cells were washed with HBSS, harvested with 0.25% Trypsin, and resuspended in 100µL 1x PBS. 10µL of each sample was mixed with 10µL 0.4% Trypan Blue, then loaded onto a Countess Cytometer to calculate the percentage of $Trypan^{+}$ cells. Biological triplicates for each condition (Control vs PFI-3) were included, and each sample was measured in duplicate to calculate an average for the final data point. A two-tailed Student's t-test was used to analyze the data.

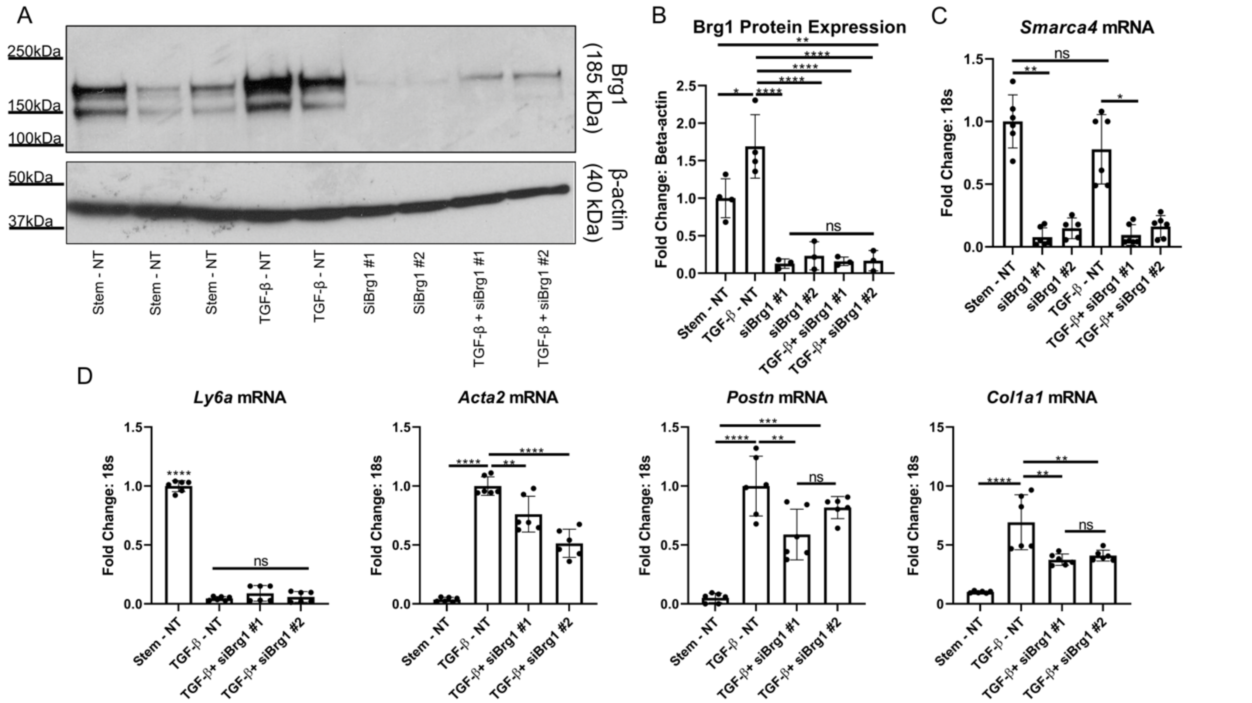

Supplemental Figure IX. siRNA-mediated knockdown of Brg1 blunts TGF-β induced gene expression of fibrosis-related genes. (A). Subcultured AdvSca1-SM cells were transfected with 100nM of scramble non-targeting control shRNA ("NT") or with one of two independent siRNA constructs targeting Brg1 ("siBrg1 #1" or "siBrg1 #2") for 48 hours, and then cultured in stem-cell media ("Stem") or stimulated with 5ng/mL TGF-β_1 ("TGF-β"), for 72 hours. Total protein lysate was harvested and subject to immunoblotting and visualized with horseradish peroxidase chemiluminescence. **(B).** Quantification of Brg1 protein normalized to β-actin. Data are pooled from at least three independent experiments. A one-way ANOVA with Tukey's multiple comparisons test was performed to compare the means of the experimental groups; **P*<0.05, **P<0.01, ****P<0.0001. **(C).** Subcultured AdvSca1-SM cells were transfected with 100nM of scramble non-targeting control shRNA ("NT") or with one of two independent siRNA constructs targeting Brg1 ("siBrg1 #1" or "siBrg1 #2") for 48 hours, and then cultured in stem-cell media ("Stem") or stimulated with 5ng/mL TGF-β_1 ("TGF-β"), for 72 hours. RNA was harvested for quantitative reverse transcription polymerase chain reaction (qRT-qPCR) analysis. Data are pooled from three independent experiments. A Kruskal-Wallis with Dunn's multiple comparisons test was performed to compare the medians of the experimental groups; *P<0.05, ***P*<0.01. **(D).** Subcultured AdvSca1-SM cells were transfected with 100nM of scramble non-targeting control shRNA ("NT") or with one of two independent siRNA constructs targeting Brg1 ("siBrg1 #1" or "siBrg1 #2") for 48 hours, and then cultured in stem-cell media ("Stem"), or stimulated with 5ng/mL TGF-β_1 ("TGF-β"), for 24 hours. RNA was harvested for qRT-qPCR analysis. *Postn* expression was measured at 72 hours. Data represent six individual biological replicates. A one-way ANOVA with Tukey's multiple comparisons test was performed to compare the means of the experimental groups; ***P*<0.01, ****P*<0.001, ****P<0.0001.

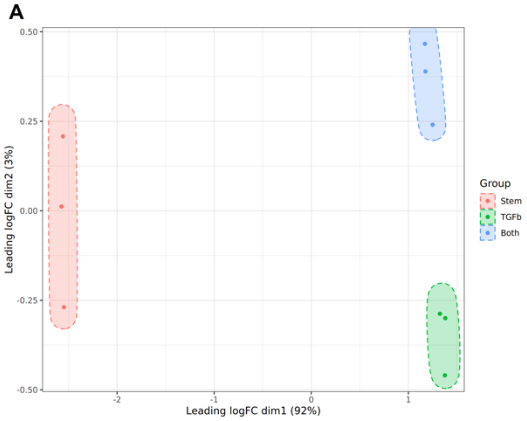

B

Pathway analysis of genes upregulated by TGF-β compared to stem

Pathway	Database	q-value
Extracellular matrix organization	Reactome	4.8e-12
Collagen formation	Reactome	4.8e-12
Metabolism	Reactome	1.02e-09
Collagen chain trimerization	Reactome	4.03e-08
Collagen biosynthesis and modifying enzymes	Reactome	4.03e-08
Human papillomavirus infection	KEGG	3.95e-07
Proteoglycans in cancer	KEGG	4.41e-07
Assembly of collagen fibrils and other multimeric structures	Reactome	4.57e-07
ECM-receptor interaction	KEGG	5.58e-07
Transport of small molecules	Reactome	5.58e-07

Pathway analysis of genes downregulated by TGF-β compared to stem

Pathway	Database	q-value
Complement and coagulation cascades	KEGG	1.14e-08
Pathways in cancer	KEGG	4.84e-06
Signal Transduction	Reactome	1.28e-05
Rap1 signaling pathway	KEGG	0.000119
Drug metabolism – cytochrome P450	KEGG	0.000181
Activation of C3 and C5	Reactome	0.000223
Regulation of Insulin Like Growth Factor transport and uptake	Reactome	0.00056
MAPK signaling pathway	KEGG	0.00056
Complement cascade	Reactome	0.00056
Metabolism of xenobiotics by cytochrome P450	KEGG	0.00056

Supplemental Figure X. Multidimensional scaling and Pathway Analysis of RNAseq dataset. Subcultured AdvSca1-SM cells isolated from SM22α-Cre ROSA-YFP mice were cultured in stem-cell media ("Stem"), 5ng/mL TGF-β_1 ("TGF-β"), or TGF-β_1 + 50μM PFI-3 ("Both") for 72 hours. RNA was harvested for bulk RNA sequencing. **(A).** Multi-dimensional scaling analysis of the three experimental groups. **(B)**. Gene set overrepresentation analysis was performed on the differentially expressed genes between Stem vs TGF-β to interrogate pathways associated with the genes upregulated by TGF-β (upper panel) and genes downregulated by TGF-β (lower panel).

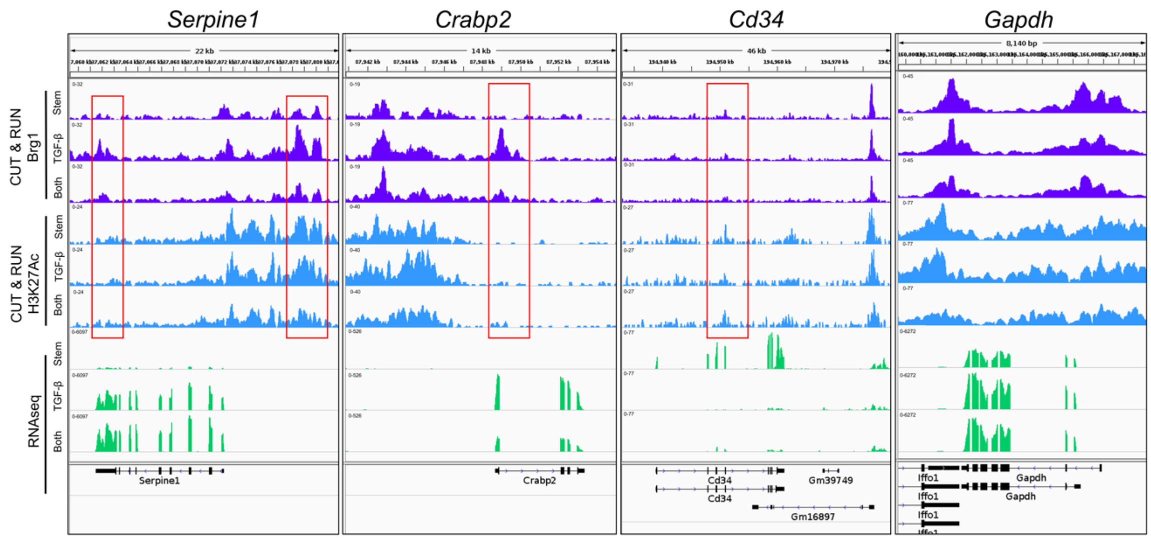

Supplemental Figure XI. IGV Plots of TGF-β inducible genes that exhibit Brg1 dependency. Subcultured AdvSca1-SM cells isolated from SM22α-Cre ROSA-YFP mice were cultured in stem-cell media (“Stem”), 5ng/mL TGF-β_1 (“TGF-β”), or TGF-β_1 + 50μM PFI-3 (“Both”) for 72 hours. Cells were adsorbed to Concavalin A beads, permeabilized, and incubated with 0.5ug of anti-Brg1 or anti-H3K27Ac antibody overnight. pAG-MNase was added to the samples to facilitate chromatin digestion and release of enriched DNA bound by Brg1. Enriched DNA was submitted for library preparation and sequenced and aligned to the mouse genome. Peaks represent enriched DNA fragments associated with Brg1 binding. IGV Plots of *Serpine1* and *Crabp2* demonstrate high degree of upregulation after TGF-β_1 stimulation by RNAseq and corresponding enrichment of Brg1 occupancy. IGV Plot of *Cd34* demonstrates high degree of downregulation after TGF-β_1 stimulation by RNAseq and corresponding decrease of Brg1 occupancy. IGV Plot of *Gapdh* demonstrates no significant change in expression after TGF-β_1 stimulation by RNAseq and no significant change in Brg1 occupancy.

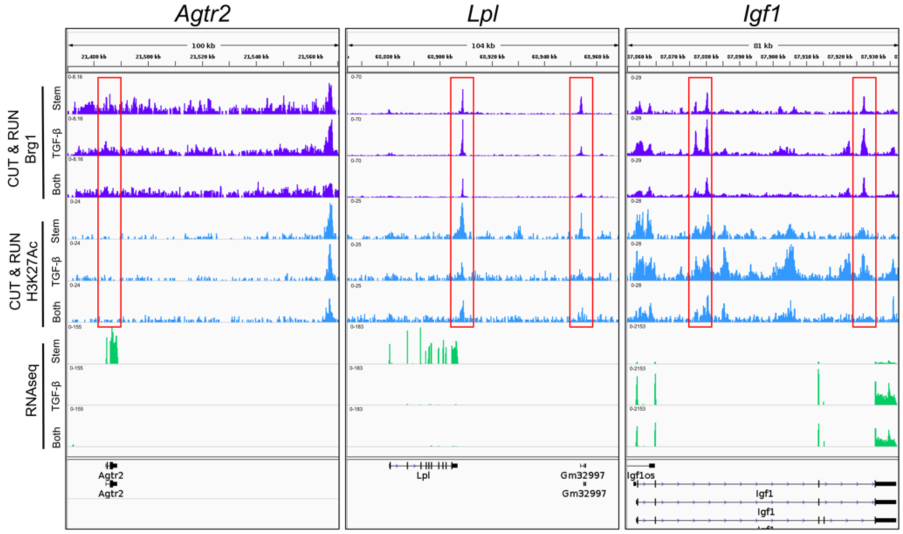

Supplemental Figure XII. IGV Plots of TGF-β inducible genes that exhibit Brg1 independency. Subcultured AdvSca1-SM cells isolated from SM22α-Cre ROSA-YFP mice were cultured in stem-cell media ("Stem"), 5ng/mL TGF-β_1 ("TGF-β"), or TGF-β_1 + 50μM PFI-3 ("Both") for 72 hours. Cells were adsorbed to Concavalin A beads, permeabilized, and incubated with 0.5ug of anti-Brg1 or anti-H3K27Ac antibody overnight. pAG-MNase was added to the samples to facilitate chromatin digestion and release of enriched DNA bound by Brg1. Enriched DNA was submitted for library preparation and sequenced and aligned to the mouse genome. Peaks represent enriched DNA fragments associated with Brg1 binding. IGV Plots of *Agtr2* and *Lpl* demonstrate high degree of downregulation after TGF-β_1 stimulation by RNAseq but no significant change in Brg1 occupancy. IGV Plot of *Igf1* demonstrates high degree of upregulation after TGF-β_1 stimulation by RNAseq but no significant change in Brg1 occupancy.

A

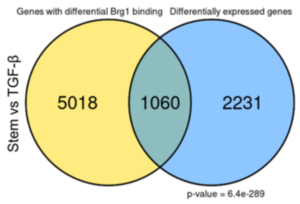

C

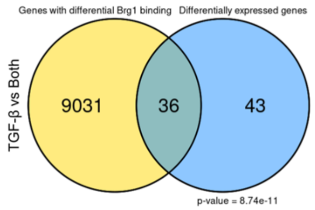

B

Unique to CUT&RUN

Pathway	Database	q-value
Signal Transduction	**Reactome**	0.00151
Oocyte meiosis	**KEGG**	0.00302
Oxytocin signaling pathway	**KEGG**	0.00302
CLEC7A (Dectin-1) signaling	**Reactome**	0.00302
RHO GTPase cycle	**Reactome**	0.00302
Longevity regulating pathway	**KEGG**	0.00302
Intracellular signaling by second messengers	**Reactome**	0.00411
Cilium assembly	**Reactome**	0.00411
C-type lectin receptor signaling pathway	**KEGG**	0.00934
cGMP-PKG signaling pathway	**KEGG**	0.00934

Unique to RNAseq

Pathway	Database	q-value
Transport of small molecules	**Reactome**	2.59e-05
Metabolism	**Reactome**	2.59e-05
Collagen formation	**Reactome**	0.000373
Complement and coagulation cascades	**KEGG**	0.000846
Glutathione conjugation	**Reactome**	0.000846
Pathways in cancer	**KEGG**	0.00138
Metabolism of amino acids and derivatives	**Reactome**	0.00138
Iron uptake and transport	**Reactome**	0.00138
Transferrin endocytosis and recycling	**Reactome**	0.00138
Fluid shear stress and atherosclerosis	**KEGG**	0.00159
Assembly of collagen fibrils and other multimeric structures	**Reactome**	0.00167

D

Unique to CUT&RUN

Pathway	Database	q-value
Signal transduction	**Reactome**	9.8e-14
Cell cycle	**Reactome**	1.03e-09
Metabolism of proteins	**Reactome**	2.17e-08
Pathways in cancer	**KEGG**	2.17e-08
Gene expression (Transcription)	**Reactome**	2.75e-08
Post-translational protein modification	**Reactome**	6.57e-08
RHO GTPase cycle	**Reactome**	1.04e-07
Intracellular signaling by second messengers	**Reactome**	6.53e-07
DNA replication	**Reactome**	6.53e-07
Cell cycle, Mitotic	**Reactome**	7.59e-07
Signaling by Receptor Tyrosine Kinases	**Reactome**	7.59e-07

Unique to RNAseq

Pathway	Database	q-value
Peptide ligand-binding receptors	**Reactome**	0.0165
ECM proteoglycans	**Reactome**	0.0306
Class A/1 (Rhodopsin-like receptors)	**Reactome**	0.0326
GPCR ligand binding	**Reactome**	0.06

Supplemental Figure XIII. Pathway analysis of non-overlapping genes in CUT&RUN and RNAseq datasets. Comparative analysis of the differential Brg1 binding from CUT&RUN versus differential gene expression from RNAseq and Metascape pathway analysis of the non-overlapping genes in the Stem vs. TGF-β and TGF-β vs. Both pairwise comparisons with the parameters of Adj. P-value <0.05 and two-fold change.

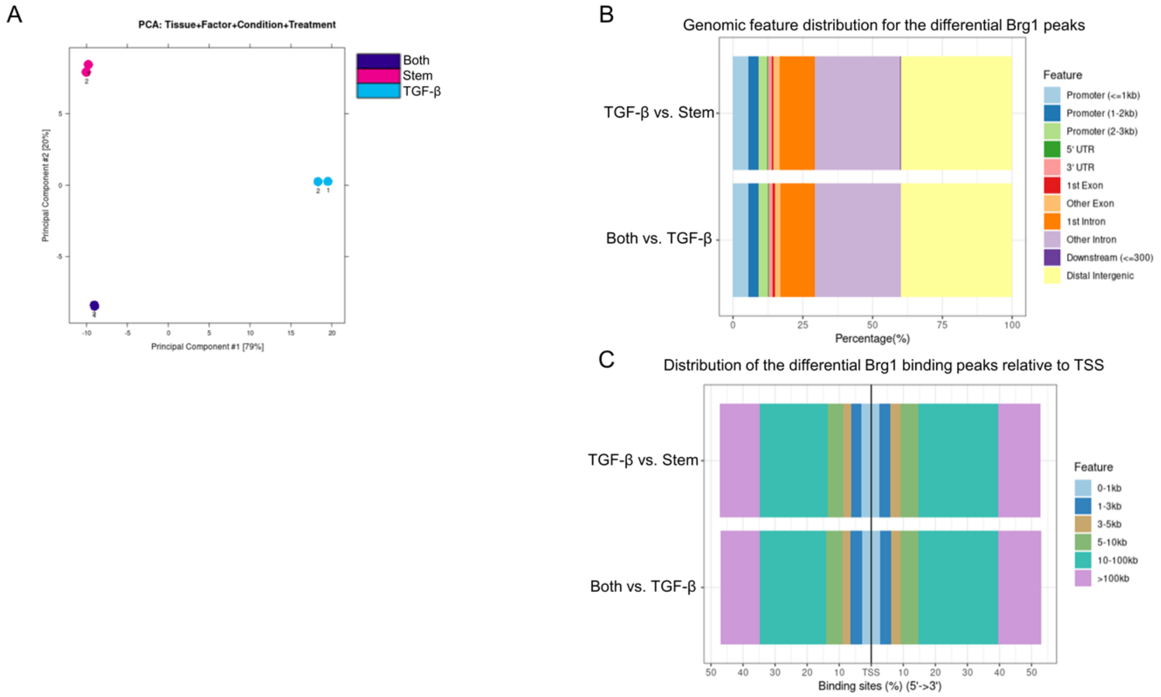

Supplemental Figure XIV. Principle Component and Genomic Enrichment Analysis differential Brg1 peaks from the CUT&RUN dataset. Subcultured AdvSca1-SM cells isolated from SM22α-Cre ROSA-YFP mice were cultured in stem-cell media (“Stem”), 5ng/mL TGF-β_1 (“TGF-β”), or TGF-β_1 + 50μM PFI-3 (“Both”) for 72 hours. Cells were adsorbed to Concavalin A beads, permeabilized, and incubated with 0.5ug of anti-Brg1 antibody overnight. pAG-MNase was added to the samples to facilitate chromatin digestion and release of enriched DNA bound by Brg1. Enriched DNA was submitted for library preparation and sequenced and aligned to the mouse genome. **(A).** Principle Component Analysis of the genes demonstrating differential Brg1 binding for “Stem”, “TGF-β”, and “Both”. **(B).** Genomic Enrichment analysis of differential Brg1 binding for Stem vs. TGF-β and TGF-β vs. Both with the parameters of Adj. P-value <0.05 and two-fold change. **(C).** Brg1 binding analysis relative to the Transcription Start Site (TSS) for Stem vs. TGF-β and TGF-β vs. Both with the parameters of Adj. P-value <0.05 and two-fold change.

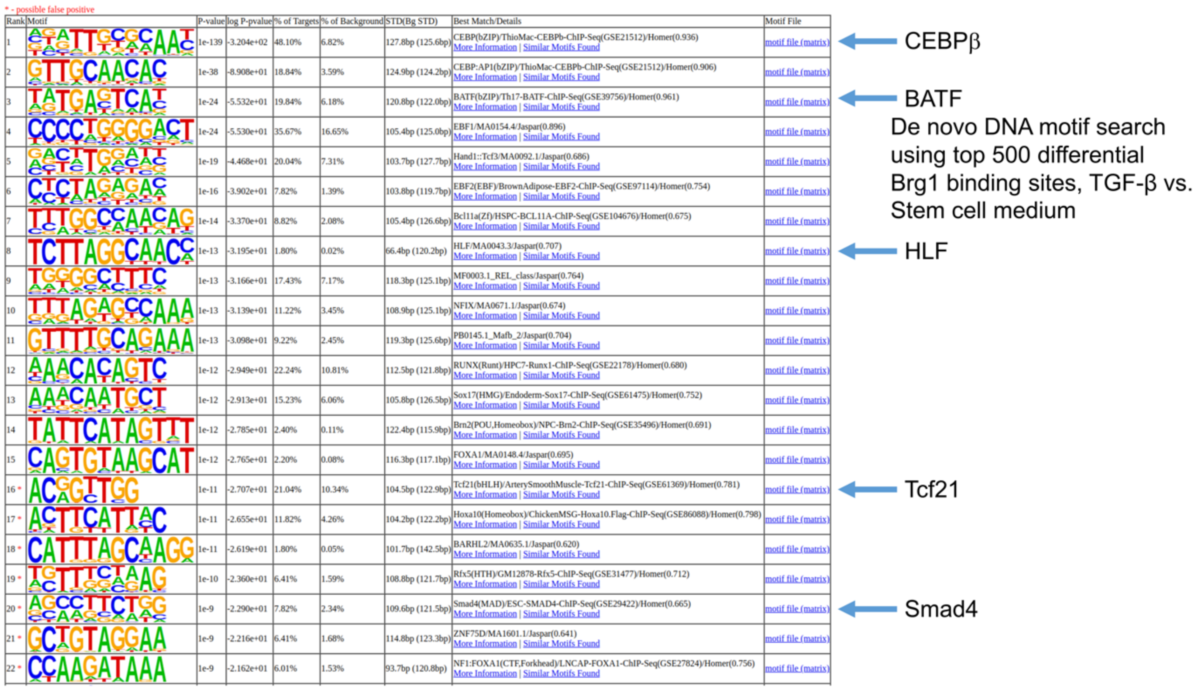

Rank	Motif	P-value	log P-pvalue	% of Targets	% of Background	STD(Bg STD)	Best Match/Details	Motif File
1		1e-139	-3.204e+02	48.10%	6.82%	127.8bp (125.6bp)	CEBP(bZIP)/ThioMac-CEBPb-ChIP-Seq(GSE21512)/Homer(0.936) More Information | Similar Motifs Found	motif file (matrix)
2		1e-38	-8.908e+01	18.84%	3.59%	124.9bp (124.2bp)	CEBP:AP1(bZIP)/ThioMac-CEBPb-ChIP-Seq(GSE21512)/Homer(0.906) More Information | Similar Motifs Found	motif file (matrix)
3		1e-24	-5.532e+01	19.84%	6.18%	120.8bp (122.0bp)	BATF(bZIP)/Th17-BATF-ChIP-Seq(GSE39756)/Homer(0.961) More Information | Similar Motifs Found	motif file (matrix)
4		1e-24	-5.530e+01	35.67%	16.65%	105.4bp (125.0bp)	EBF1/MA0154.4/Jaspar(0.896) More Information | Similar Motifs Found	motif file (matrix)
5		1e-19	-4.468e+01	20.04%	7.31%	103.7bp (127.7bp)	Hand1::Tcf3/MA0092.1/Jaspar(0.686) More Information | Similar Motifs Found	motif file (matrix)
6		1e-16	-3.902e+01	7.82%	1.39%	103.8bp (119.7bp)	EBF2(EBF)/BrownAdipose-EBF2-ChIP-Seq(GSE97114)/Homer(0.754) More Information | Similar Motifs Found	motif file (matrix)
7		1e-14	-3.370e+01	8.82%	2.08%	105.4bp (126.6bp)	Bcl11a(Zf)/HSPC-BCL11A-ChIP-Seq(GSE104676)/Homer(0.675) More Information | Similar Motifs Found	motif file (matrix)
8		1e-13	-3.195e+01	1.80%	0.02%	66.4bp (120.2bp)	HLF/MA0043.3/Jaspar(0.707) More Information | Similar Motifs Found	motif file (matrix)
9		1e-13	-3.166e+01	17.43%	7.17%	118.3bp (125.1bp)	MF0003.1_REL_class/Jaspar(0.764) More Information | Similar Motifs Found	motif file (matrix)
10		1e-13	-3.139e+01	11.22%	3.45%	108.9bp (125.1bp)	NFIX/MA0671.1/Jaspar(0.674) More Information | Similar Motifs Found	motif file (matrix)
11		1e-13	-3.098e+01	9.22%	2.45%	119.3bp (125.6bp)	PB0145.1_Mafb_2/Jaspar(0.704) More Information | Similar Motifs Found	motif file (matrix)
12		1e-12	-2.949e+01	22.24%	10.81%	112.5bp (121.8bp)	RUNX(Runt)/HPC7-Runx1-ChIP-Seq(GSE22178)/Homer(0.680) More Information | Similar Motifs Found	motif file (matrix)
13		1e-12	-2.913e+01	15.23%	6.06%	105.8bp (126.5bp)	Sox17(HMG)/Endoderm-Sox17-ChIP-Seq(GSE61475)/Homer(0.752) More Information | Similar Motifs Found	motif file (matrix)
14		1e-12	-2.785e+01	2.40%	0.11%	122.4bp (115.9bp)	Brn2(POU,Homeobox)/NPC-Brn2-ChIP-Seq(GSE35496)/Homer(0.691) More Information | Similar Motifs Found	motif file (matrix)
15		1e-12	-2.765e+01	2.20%	0.08%	116.3bp (117.1bp)	FOXA1/MA0148.4/Jaspar(0.695) More Information | Similar Motifs Found	motif file (matrix)
16 *		1e-11	-2.707e+01	21.04%	10.34%	104.5bp (122.9bp)	Tcf21(bHLH)/ArterySmoothMuscle-Tcf21-ChIP-Seq(GSE61369)/Homer(0.781) More Information | Similar Motifs Found	motif file (matrix)
17 *		1e-11	-2.655e+01	11.82%	4.26%	104.2bp (122.2bp)	Hoxa10(Homeobox)/ChickenMSG-Hoxa10.Flag-ChIP-Seq(GSE86088)/Homer(0.798) More Information | Similar Motifs Found	motif file (matrix)
18 *		1e-11	-2.619e+01	1.80%	0.05%	101.7bp (142.5bp)	BARHL2/MA0635.1/Jaspar(0.620) More Information | Similar Motifs Found	motif file (matrix)
19 *		1e-10	-2.360e+01	6.41%	1.59%	108.8bp (121.7bp)	Rfx5(HTH)/GM12878-Rfx5-ChIP-Seq(GSE31477)/Homer(0.712) More Information | Similar Motifs Found	motif file (matrix)
20 *		1e-9	-2.290e+01	7.82%	2.34%	109.6bp (121.5bp)	Smad4(MAD)/ESC-SMAD4-ChIP-Seq(GSE29422)/Homer(0.665) More Information | Similar Motifs Found	motif file (matrix)
21 *		1e-9	-2.216e+01	6.41%	1.68%	114.8bp (123.3bp)	ZNF75D/MA1601.1/Jaspar(0.641) More Information | Similar Motifs Found	motif file (matrix)
22 *		1e-9	-2.162e+01	6.01%	1.53%	93.7bp (120.8bp)	NF1:FOXA1(CTF,Forkhead)/LNCAP-FOXA1-ChIP-Seq(GSE27824)/Homer(0.756) More Information | Similar Motifs Found	motif file (matrix)

Supplemental Figure XV. Transcription Factor Motif Analysis of differential Brg1 peaks from the CUT&RUN dataset. The HOMER database from UCSD (v4.11) was utilized to search de novo DNA motifs using top 500 differential Brg1 binding sites comparing TGF-β vs. Stem samples. Transcription factor motifs identified are shown, including Tcf21 and Smad4, which are involved in fibrosis and TGF-β signaling (blue arrows). CEBPβ, BATF, and HLF have also been reported to be involved in fibrosis in other cell systems.

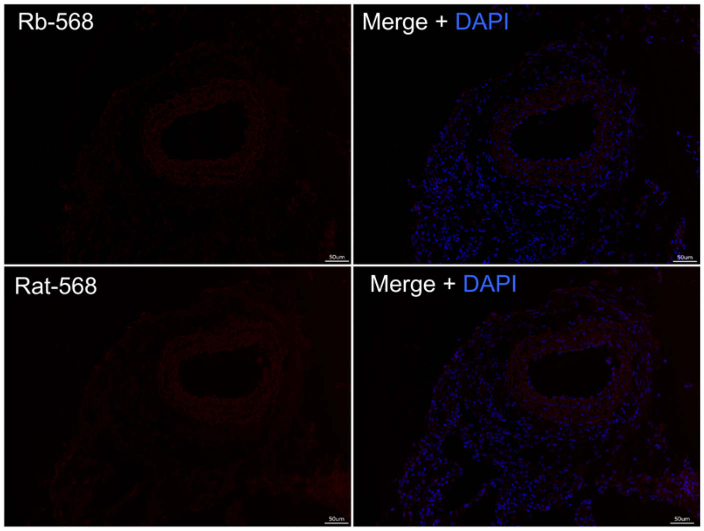

Supplemental Figure XVI: IgG isotype controls for immunofluorescence. Injured arterial sections from Gli1-Cre^{ERT2} ROSA-YFP mice were stained using rabbit (Rb) or rat IgGs followed by respective secondary antibodies as negative controls for immunofluorescence.

Uncropped/Unedited Gel Images for Western Blot Data

Mary Weiser-Evans

164862-INS-RG-TR-2

Redistribution of the chromatin remodeler Brg1 directs smooth muscle-derived adventitial progenitor-to-myofibroblast differentiation and vascular fibrosis

Full unedited gel for Supplemental Figure IX

After the transfer step from the acrylamide gel to the PVDF membrane, the PVDF membrane was cut with scissors at the 75kDa ladder mark to blot for Brg1 on the top half of the membrane and β-actin on the lower half of the membrane. This unedited image is from X-Ray film that was exposed to the chemiluminescence reaction for 30 seconds. Only the Brg1 bands were used for the final version of Supplemental Figure IX, not the β-actin bands as 30 seconds is too much exposure for β-actin.

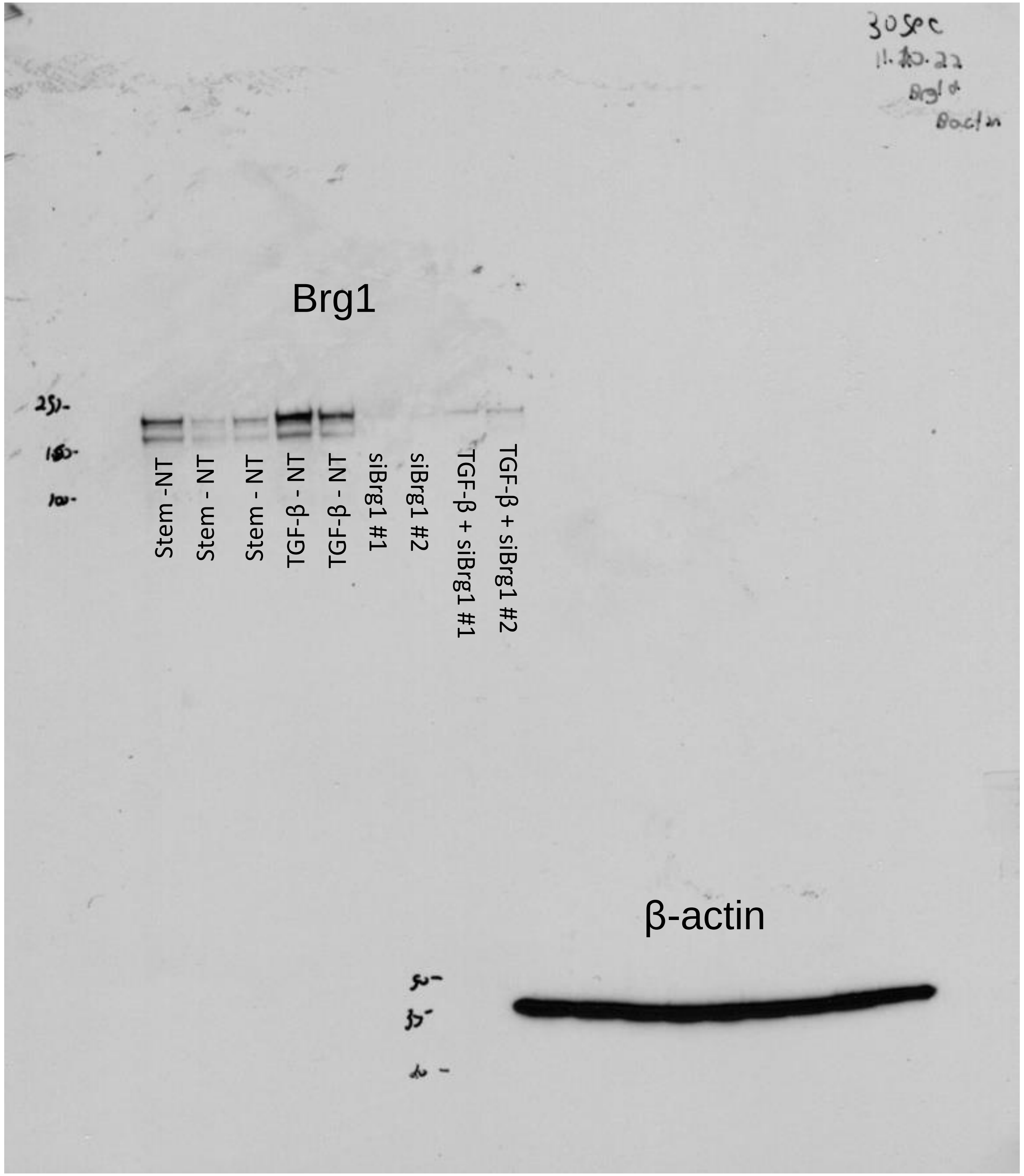

Full unedited gel for Supplemental Figure IX

After the transfer step from the acrylamide gel to the PVDF membrane, the PVDF membrane was cut with scissors at the 75kDa ladder mark to blot for Brg1 on the top half of the membrane and β-actin on the lower half of the membrane. This unedited image is from X-Ray film that was exposed to the chemiluminescence reaction for 3 seconds. Only the β-actin bands from this blot were used for the final version of Supplemental Figure IX , not the Brg1 bands 3 seconds is too little exposure to visualize Brg1.

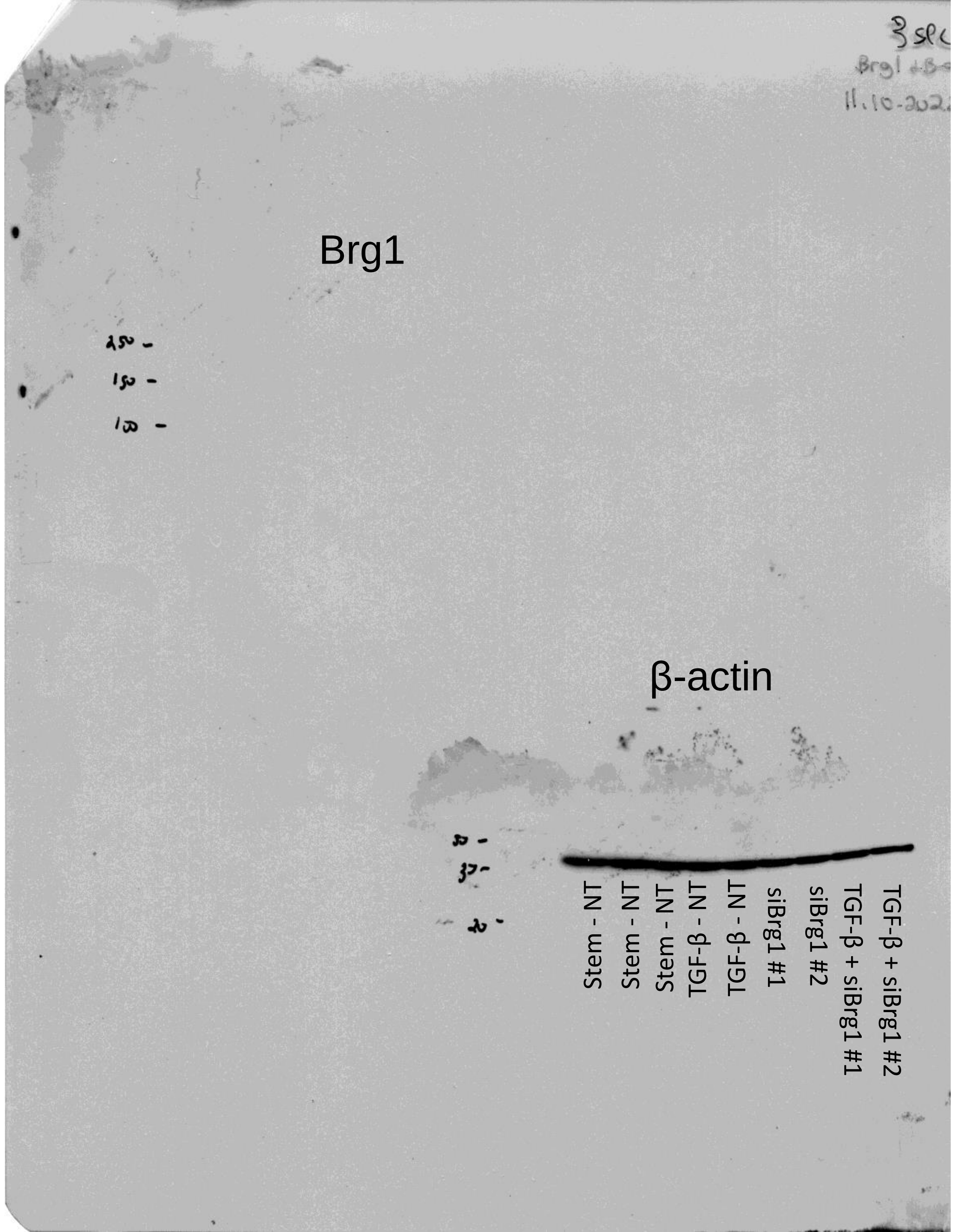

Supplement: Supplemental data [file jciinsight-8-164862-s187.pdf]
